# miR-129-3p alleviates chondrocyte apoptosis in knee joint fracture-induced osteoarthritis through CPEB1

**DOI:** 10.1186/s13018-020-02070-1

**Published:** 2020-11-23

**Authors:** Ruixiong Chen, Baoqing Ye, Han Xie, Yuliang Huang, Zhehui Wu, Hongbo Wu, Xiaofeng Wang, Haixiong Miao, Weiguo Liang

**Affiliations:** 1 Department of Orthopedics, Guangzhou Red Cross Hospital Affiliated to Jinan University, No. 396, Mid Tongfu Road, Haizhu District, Guangzhou, 510000 Guangdong People’s Republic of China; 2 Department of Orthopedics, Huizhou Central People’s Hospital, Huizhou, 516000 Guangdong People’s Republic of China

**Keywords:** miR-129-3p, CPEB1, Knee joint fracture, Osteoarthritis, Cell viability, Chondrocyte apoptosis

## Abstract

**Background:**

Osteoarthritis (OA), a refractory disease, is one of the leading contributors for disability worldwide. Since chondrocyte is the only resident cell in cartilage, this study aims to explore the roles of miR-129-3p and CPEB1 in chondrocyte apoptosis in knee joint fracture-induced OA.

**Methods:**

Cartilage was collected from 20 OA patients who underwent total knee replacement (OA group) and 20 patients with knee contusion (normal group). Then, miR-129-3p and CPEB1 levels in the cartilage were quantified by qRT-PCR. Primary rat chondrocytes in the knee were isolated and identified by toluidine blue staining and immunofluorescent staining of type II collagen. OA cellular models were induced by TNF-α treatment, in which miR-129-3p and CPEB1 expressions were assessed. Subsequently, cell viability, apoptosis, and the expression levels of apoptotic protein and caspase-3 were measured. Dual luciferase reporter assay identified the interaction between miR-129-3p and CPEB1.

**Results:**

Patients in the OA group had decreased miR-129-3p expression and increased CPEB1 expression than those in the normal group. TNF-α treatment successfully induced the OA cellular model. Downregulated miR-129-3p and upregulated CPEB1 expressions were found in OA-treated chondrocytes. miR-129-3p overexpression or CPEB1 knockdown improved chondrocyte viability and attenuated apoptosis, and vice versa. miR-129-3p negatively regulated CPEB1, thus ameliorating apoptosis and enhancing cell viability.

**Conclusion:**

miR-129-3p negatively targeted CPEB1 to facilitate chondrocyte viability and hamper apoptosis.

**Supplementary Information:**

The online version contains supplementary material available at 10.1186/s13018-020-02070-1.

## Introduction

Among the elderly over the age of 50 years, osteoarthritis (OA) is the 12th leading contributor for disability worldwide in 2017 [1]. Aging, overweight, female sex, high physical occupational load and joint injury are known risk factors for knee OA, and the development of knee OA is induced by knee injuries occurred in adolescence and young adulthood [2]. OA is a joint disease characterized by degradation of articular cartilage (AC), inflammation of synovium and joint fat pad, as well as alterations in bone structure [3]. To date, the current OA treatment is limited to pain relief or joint arthroplasty for end-staged OA [4]. Chondrocytes are the only resident cells in AC and are responsible for the generation and turnover of the extracellular matrix, which undergo the maintenance of cartilaginous function and structure [5]. Previous studies demonstrated that chondrocyte apoptosis is definitely correlated with cartilage degeneration in OA [6, 7]. Therefore, a better understanding of the molecular mechanisms underlying chondrocyte apoptosis in OA is urgently needed to facilitate OA treatment.

MicroRNAs (miRs), non-coding RNAs (~ 22 nucleotide at length) interacting with cognate messenger RNAs (mRNAs), modify posttranscriptional regulation of genes through enhancement of degradation, by suppressing translation, or via other mechanisms [8]. miRs play important roles in the regulation of chondrocyte function by interacting with targeted mRNAs associated with OA pathology [9]. miR-129-3p has been reported as a tumor suppressor with decreased expression in various types of human cancer, which could regulate many cancer-related phenotypes. For example, miR-129-3p presented as an inhibitor of glioblastoma growth, and it is regarded as a new target for the glioblastoma treatment [10]. miR-129-3p has also been mentioned in renal cell carcinoma as a promising diagnostic biomarker and a suppressor of multiple metastasis-related genes [11]. More importantly, the anti-apoptotic effect of miR-129-3p has been elucidated in cardiomyocytes and breast cancer cells [12, 13]. miR-129-3p has also been confirmed to inhibit the expression of inflammatory cytokine IL-17 in rheumatoid arthritis [14]. In addition, miR-129-5p, another mature form of miR-129, has been demonstrated to mediate the proliferation of chondrocyte in developmental dysplasia of the hip [15]. These facts provided us an insight that miR-129-3p might have a crucial role in chondrocyte apoptosis; however, no investigation has reported the action mechanisms of miR-129-3p in chondrocyte apoptosis induced by OA.

In this study, we reported here that miR-129-3p played a crucial role in chondrocyte apoptosis. Based on these results, novel insight is emerging considering the use of miR-129-3p as a possible therapeutic agent in OA.

## Materials and methods

### Ethical statement

Obtainment of clinical tissues and the design of animal experiment were approved by the ethical committee of Huizhou Central People’s Hospital. All participants provided informed consents. All efforts were made to reduce the pain of experimental animals as much as possible.

### Clinical sample

Diagnosis of knee OA was referred to the American Rheumatism Association (ARA) criteria for the classification of knee OA (Table 1). Patients diagnosed as knee joint fracture–induced OA were included into this study. Exclusion criteria were as follows: explicit trauma history in the knee; operation history; dyscrasia; history of steroid injections, tumor, and infection. A total of 20 OA patients (consisting of 12 females and 8 males, average age of 66.4 years) who underwent total knee replacement were recruited in this study to collect the cartilage at the injured site and named OA group. On the other hand, unworn cartilage was collected from 20 patients with knee contusion (normal group, consisting of 9 females and 11 males, average age of 62.6 years) who underwent unicompartmental knee arthroplasty or total knee replacement. The tissues were collected from each patient during the operation and immediately maintained in liquid nitrogen.

**Table 1:** American Rheumatism Association (ARA) criteria for the classification of knee OA

Numerical order	Clinical characteristics
1	Knee joint ache most of the time before onset of knee OA
2	Clicking of joint
3	Morning stiffness for less than 30 min
4	Over 40 years old
5	Swollen knee joint; clicking of joint
6	Swollen knee joint; without clicking of joint

In clinical criteria, knee OA was diagnosed if the patient met the criteria of 1 + 2 + 3 + 4, 1 + 2 + 3 + 5, or 1 + 6

*OA* osteoarthritis

### Cell culture

Male Wistar rats, weighting 140 ± 10 g, were obtained from a center for experimental animals, Chinese Academy of Sciences. During experimental time course, all rats were housed at 22~25 °C with 12 h light/dark cycle and were fed with food and water ad libitum. After anesthetization, the rats were killed by cervical dislocation. Cartilage in the surfaces of knee joint was separated aseptically and digested by pancreatin for 30 min at 37 °C and by 0.2% type II collagenase (Thermo, Waltham, MA, USA) for 3 h, during which the cartilage was shaken in an orbital shaker incubator (37 °C) for 5 min every hour. The cartilage tissues were observed under an inverted microscope once massive floccules can be seen by naked eyes. After most of the chondrocytes were separated, the chondrocytes were flapped by a pipette tip (1 ml). The digestion was terminated using fetal bovine serum (FBS, Gibco, Carlsbad, CA). Then, the cells were filtered (200 mesh) and centrifuged (1500 r/min, 10 min), followed by PBS washes 3 times. The cells were collected and incubated with DMEM (Thermo, Waltham, MA, USA) supplemented with 10% FBS and 1% penicillin/streptomycin (Solarbio, Beijing, China) in an incubator (Thermo, Waltham, MA, USA) under 5% CO_2_ at 37 °C. After 48 h of incubation, the culture medium was replaced, and the non-adhered cells were removed. Thereafter, the culture medium was replaced every other day. An inverted microscope was used to record the morphology and adherence of the cells. Subculture was performed when the adhered cells were at the proportion of 85~90%. The 3rd~5th generation was utilized for the following experiments and was identified by toluidine blue staining and type II collagen staining.

### Toluidine blue staining

Well-adhered chondrocytes were digested for preparing cell suspension and inoculated into 6-well plates at a density of 1 × 10^5^/mL. The cells were cultured in an incubator until the cells were fully adhered, and the confluence of these cells was more than 80%. The culture medium was removed before the cells were washed with PBS for three times and blotted. The cells in each well were fixed with 2 mL of 4% paraformaldehyde in a 4 °C refrigerator for 1 h, after which the cells were washed with PBS for three times and were blotted. Toluidine blue (1 mL, Sangon Biotech, Shanghai, China) was added for staining at room temperature. After 1 h of staining, the toluidine blue was discarded. The cells were washed with PBS for three times to remove floating color. After being blotted, the cells were observed and photographed under an inverted microscope.

### Immunofluorescent staining of type II collagen

Well-adhered chondrocytes were digested, and cell suspension was prepared. Then, the chondrocytes were inoculated into a disposable culture dish at the density of 1 × 10^5^/mL and incubated. After the cells were adhered, the culture medium was discarded, and the chondrocytes were washed with PBS for 3 times. After the chondrocytes were blotted, 2 mL of 4% paraformaldehyde was added into the culture dish. The chondrocytes were placed for 30 min at room temperature and washed with PBS for 3 times before being blotted by filter paper. The culture dish was added with 1 mL of 0.1% Triton X-100 solution for 20 min of incubation, followed by PBS washes 3 times. Then, the chondrocytes were blotted and blocked with 10% goat serum (1 mL) for 30 min. After that, the chondrocytes were blotted but not rinsed. Goat serum (10%, ZSGB-BIO, Beijing, China)-diluted rabbit anti-type II collagen (1 mL, ab34712, 1:200, Abcam, MA, USA) was incubated with the chondrocytes at 4 °C overnight in the dark. The chondrocytes were subjected to PBS washes 3 times before being incubated with 1 mL of fluorescent secondary antibody (ab150077, 1:500, Abcam, MA, USA) diluted by 10% goat serum at 37 °C for 1 h. DAPI solution (1 mL, ab104139, 1:1000, Abcam, MA, USA) was added for staining at room temperature for 2 min after the culture dish was rinsed with PBS and blotted with filter paper. After being washed with PBS for three times, the culture dish was added with 1 mL of anti-fade mounting agent and was visualized under a confocal laser scanning microscope (green wavelength 543 nm, blue wavelength 458 nm, × 200).

### OA cellular model

TNF-α (20 ng/ml, PeproTech, Rocky Hill, NJ, USA) was used to establish OA cellular model. The chondrocytes used for OA model were grouped into OA group, and the chondrocytes subjected to routine culture were grouped into sham group. In brief, TNF-α (20 ng/ml) was added into the culture medium of chondrocytes for 6 h of incubation; after which, the chondrocytes were successively cultured for 24 h in normal culture medium. After that, the cell performance and the expression levels of miR-129-3p and CPEB1 were detected. To study the influence of miR-129-3p and CPEB1 on OA-induced cells, the cells in the 6-well plates were transfected with corresponding plasmids and then cultured in the normal culture medium, followed by incubation with TNF-α (20 ng/ml) for 6 h and with normal culture medium for 24 h. Subsequently, cell performance and expression levels of miRNA and protein were measured.

### Cell transfection and grouping

miR-129-3p mimic (100 nM), mimic NC (100 nM), miR-129-3p inhibitor (100 nM), inhibitor NC (100 nM), pcDNA3.1-CPEB1 (2 μg), and pcDNA3.1 (2 μg) were purchased from Shanghai GenePharma Co., Ltd (Shanghai, China). The cells in the 6-well plates at 70~80% confluence were transfected with miR-129-3p mimic, mimic NC, miR-129-3p inhibitor, inhibitor NC, pcDNA3.1, or pcDNA3.1-CPEB1 using Lipofectamine 2000 reagent (Invitrogen, Carlsbad, CA, USA) in accordance with the directions. The cells were correspondingly named as miR-129-3p mimic group, mimic NC group, miR-129-3p inhibitor group, inhibitor NC group, pcDNA3.1 group, or pcDNA3.1-CPEB1 group. After 48 h of transfection, the cells were used for the following experiments.

### qRT-PCR

Total RNA was extracted from cultured chondrocytes and tissues using TRIZOL (Invitrogen, Carlsbad, CA, USA). cDNA was synthesized using reverse transcription kit (TaKaRa, Tokyo, Japan) following the instructions. qRT-PCR was performed using LightCycler 480 (Roche, Indianapolis, IN, USA) in accordance with the protocols of fluorescent qPCR kit (SYBR Green Mix, Roche Diagnostics, Indianapolis, IN). The amplifications were carried out at 95 °C for 10 s, followed by 45 cycles of 95 °C for 5 s, 60 °C for 10 s, and 72° C for 10 s, and then were ended with 5 min of extension at 72 °C. Each qPCR was performed for three times. GAPDH and U6 were applied for normalization. Data was analyzed by 2^−ΔΔCt^ method. ΔΔCt = experimental group (Ct _target gene_ − Ct _internal control_) − control group (Ct _target gene_ − Ct _internal control_). The primer sequences are listed in Table 2.

**Table 2:** Primer sequences for qRT-PCR

Name of primer		Sequences (5′-3′)
miR-129-3p	Forward	AAGCCCTTACCCCAAAAAGTAT
	Reverse	CTTTTTGCGGTCTGGGCTTGC
U6	Forward	UUCUCCGAACGUGUCACGUTT
	Reverse	UGACACGUUCGGAGAATT
CPEB1	Forward	GATGCAAATGACTTGTGCCTTG
	Reverse	GGCTGAGGAATCTGAGTCCTG
GAPDH	Forward	GGAGCGAGATCCCTCCAAAAT
	Reverse	GGCTGTTGTCATACTTCTCATGG

### Western blot

Total proteins were obtained from chondrocytes by using RIPA lysis buffer (Beyotime, Shanghai, China) and quantified by BCA kit (Beyotime). The corresponding proteins and the loading buffer (Beyotime) were mixed and then denatured by using boiling water bath for 5 min. Then, the proteins were subjected to electrophoresis at 80 V for 30 min which were switched to 120 V for 1~2 h when bromophenol blue entered into separation gel. Following separation, membrane transferring was measured by using ice bath at 220 mA for 120 min. Subsequently, the membrane was rinsed in wash buffer for 1~2 min, and then were blocked in blocking buffer at room temperature for 60 min. Primary antibodies against GAPDH (5174S, 1:1000, Cell Signaling, Boston, USA), CPEB1 (ab3465, 1:1000, Abcam, MA, USA), and cleaved caspase-3 (ab49822, 1:1000, Abcam, MA, USA) were added and incubated with the membranes at 4 °C overnight, followed by washes with wash buffer for 3 × 10 min the next day. Afterwards, the membrane was incubated with secondary antibody (horseradish peroxidase-labeled goat anti rabbit IgG, 1:5000, Beijing ComWin Biotech Co., Ltd, Beijing, China) at room temperature for 1 h and was washed for 3 × 10 min. After being developed, the bands were visualized by a chemiluminescence imaging system (Bio-rad).

### Flow cytometry

Six-well plates were used to prepare 1 ml of cell suspension (5 × 10^5^ cells/well) and pre-cultured in an incubator gassed with 5% CO_2_ at 37 °C for 24 h. Then, the adhered cells were digested by pancreatin and washed by pre-cooled PBS twice (2000 rpm, 5 min). Annexin V binding buffer (1 mL) was added to resuspend the cells, and 10 μl of propidium iodide (PI) and 5 μl of annexin V-FITC were successively added and incubated with the cells for 30 min in the dark. One hour later, the cells were detected by flow cytometry. Each test was performed for three times.

### CCK8

Ninety-six-well plates were exploited for the preparation of cell suspension (100 μl/well) at a density of 1500 cells/well. The plates were pre-cultured in an incubator at 37 °C under 5% CO_2_ for respectively 24 h, 48 h, 72 h, and 96 h, and were added with 10 μl of CCK8 solution per well. Generation of bubbles was carefully avoided during the operations. The plates were replaced into the incubator for 2 h of incubation. Subsequently, optical density (OD) value was determined by a microplate reader at 450 nm. Each test was performed in triplicate. Cell viability (%) = [A (transfection group) − A (background)]/[A (Blank group) − A (background)] × 100. A (transfection group) equaled to the OD value of the wells containing transfected cells and CCK8 solution, A (background) to the OD value of the wells with only culture solution and CCK8 solution, and A (blank group) to the OD value of the wells that contained untransfected cells and CCK8 solution.

### Dual luciferase reporter assay

Potential binding sites between miR-129-3p and CPEB1 were predicted by TargetScan (http://www.targetscan.org/vert_72/). According to the prediction, mut-CPEB1 and wt-CPEB1 were designed and synthesized. Then, luciferase reporter vectors (pGL3-Basic) were inserted with these sequences and were co-transfected with miR-129-3p mimic (0, 150 nM, 300 nM, Shanghai GenePharma Co., Ltd) into HEK293T cells. After being mixed, the cells were lysed by 100 μl of lysis buffer in a shaking bed at room temperature for 20 min. Lysed cell suspension (50 μl) was collected and added with 50 μl of luciferase solution (Promega, Madison, WI, USA) for detection of firefly luciferase activity. Stop&Glo (50 μl, Promega, USA) was added and mixed for assessment of Renilla luciferase activity which was taken as the internal control. Relative luciferase activity was defined as the ratio of firefly luciferase activity and Renilla luciferase activity. Each sample was repeated for three times.

### Statistical analysis

All experiments above were repeated for three times. Quantitative analysis was carried out using GraphPad Prism 5 (GraphPad Software Inc., San Diego, CA, USA). All data were analyzed by SPSS 12.0 (SPSS Inc., Chicago, IL, USA). Comparisons between experimental groups and control groups were assessed by one-way analysis of variance, followed by Dunnett’s test. *P* < 0.05 was regarded as statistically significant.

## Results

### Aberrant expression of miR-129-3p and CPEB1 in the cartilage from fractured knee joint

**Fig. 1:**
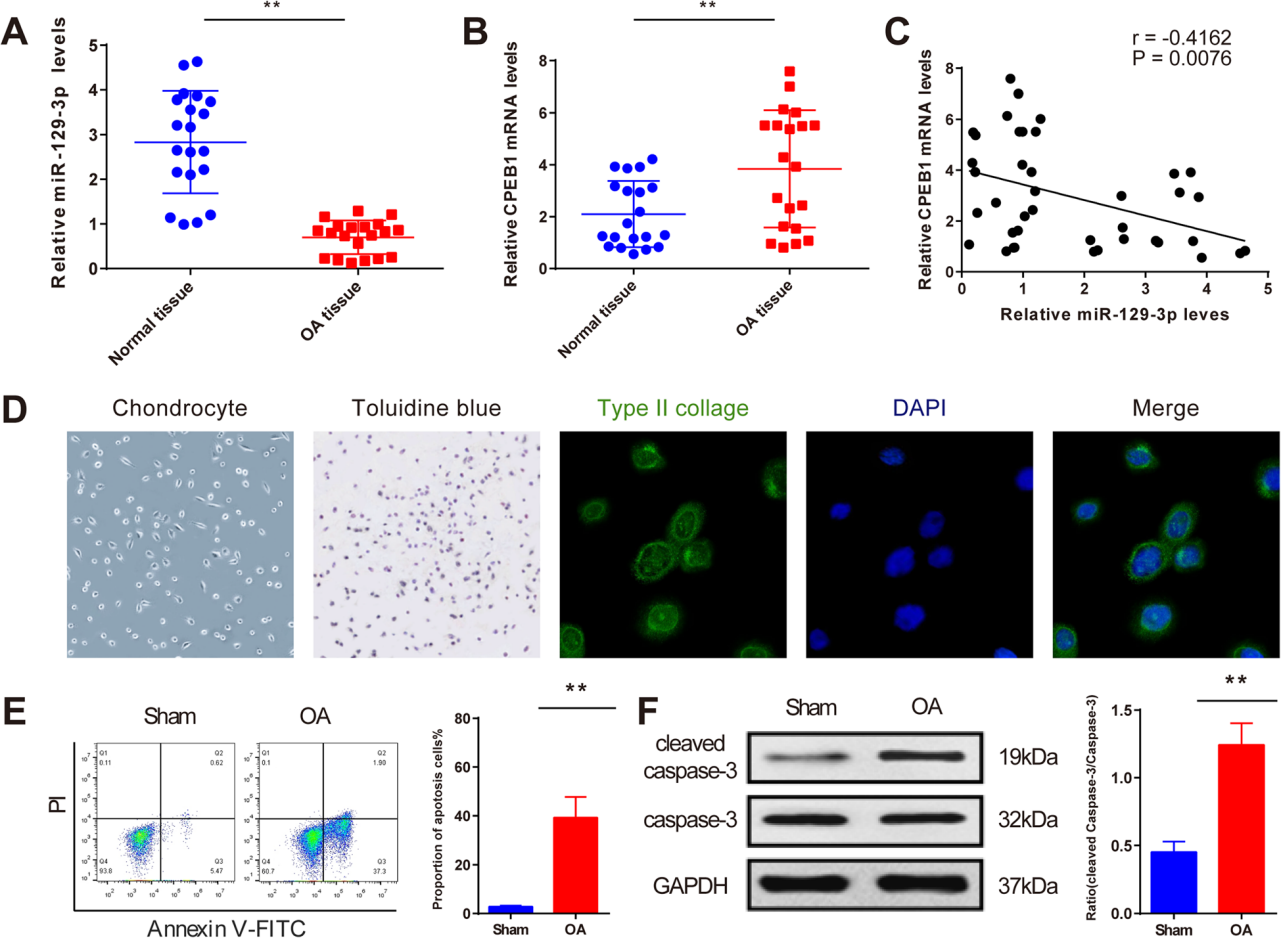
Identification of primary chondrocytes and OA cellular model. Expression levels of miR-129-3p and CPEB1 in cartilage were determined by qRT-PCR analysis (**a**, **b**); correlation analysis of miR-129-3p with CPEB1 (**c**); the morphology of chondrocytes (× 100), results of toluidine blue staining (purple, × 100), immunofluorescent staining of type II collagen (green, × 200), DAPI staining (blue, × 200), and the merge graph (× 200) (**d**). After TNF-α treatment, flow cytometry and Western blot were applied to detect chondrocyte apoptosis and cleaved caspase-3 expression, respectively (**e**, **f**). **P < 0.01. OA, osteoarthritis

qRT-PCR analysis quantified the expression levels of miR-129-3p and CPEB1 both in OA and normal groups. The results suggested that miR-129-3p expression was decreased, and mRNA expression of CPEB1 was increased in the OA group, compared to the normal group (Fig. 1a, b, *P* < 0.01). Correlation analysis suggested that miR-129-3p expression was negatively correlated with CPEB1 (Fig. 1c, *P* < 0.01). These results revealed that miR-129-3p and CPEB1 were dysregulated in OA and were negatively associated with each other, indicating the implication of miR-129-3p and CPEB1 in OA.

### Identification of rat chondrocytes and OA cellular model

Under the inverted microscope, normal chondrocytes were cultured as monolayers and adherently expanded in irregularly circular or polygonal shape and overspread the bottle bottom (Fig. 1d). Toluidine blue stained chondrocyte-derived aggrecan and found that the cell membranes and cytoplasm of chondrocytes were blueviolet, and the nuclei were dark purple with obvious nucleolus (Fig. 1d). Immunofluorescent staining of type II collagen for the 2nd generation demonstrated that the cytoplasm was green after anti-type II collagen-specific antibody was added, and the nuclei was blue after DAPI was added (Fig. 1d), suggesting that the separated cells had the properties of chondrocytes. Taken the results of morphological observation, toluidine blue staining, immunofluorescent staining of type II collagen, and the sites we collected the cells into consideration, it can be concluded that the cells we separated were chondrocytes in the knee joint.

In this study, TNF-α was used to induce OA cellular model to mimic OA induced by knee joint fracture, and OA cellular model was identified by flow cytometry and Western blot. According to the results of flow cytometry and Western blot, in the OA group, chondrocyte apoptosis was upregulated as well as the expression of cleaved caspase-3 compared to the Sham group (Fig. 1e, f, *P* < 0.01). Taken together, the separated primary cells were chondrocytes in the knee joint, and TNF-α could induce chondrocyte apoptosis, suggesting a successful establishment of OA cellular model.

### miR-129-3p attenuates chondrocyte apoptosis in OA cellular model

**Fig. 2:**
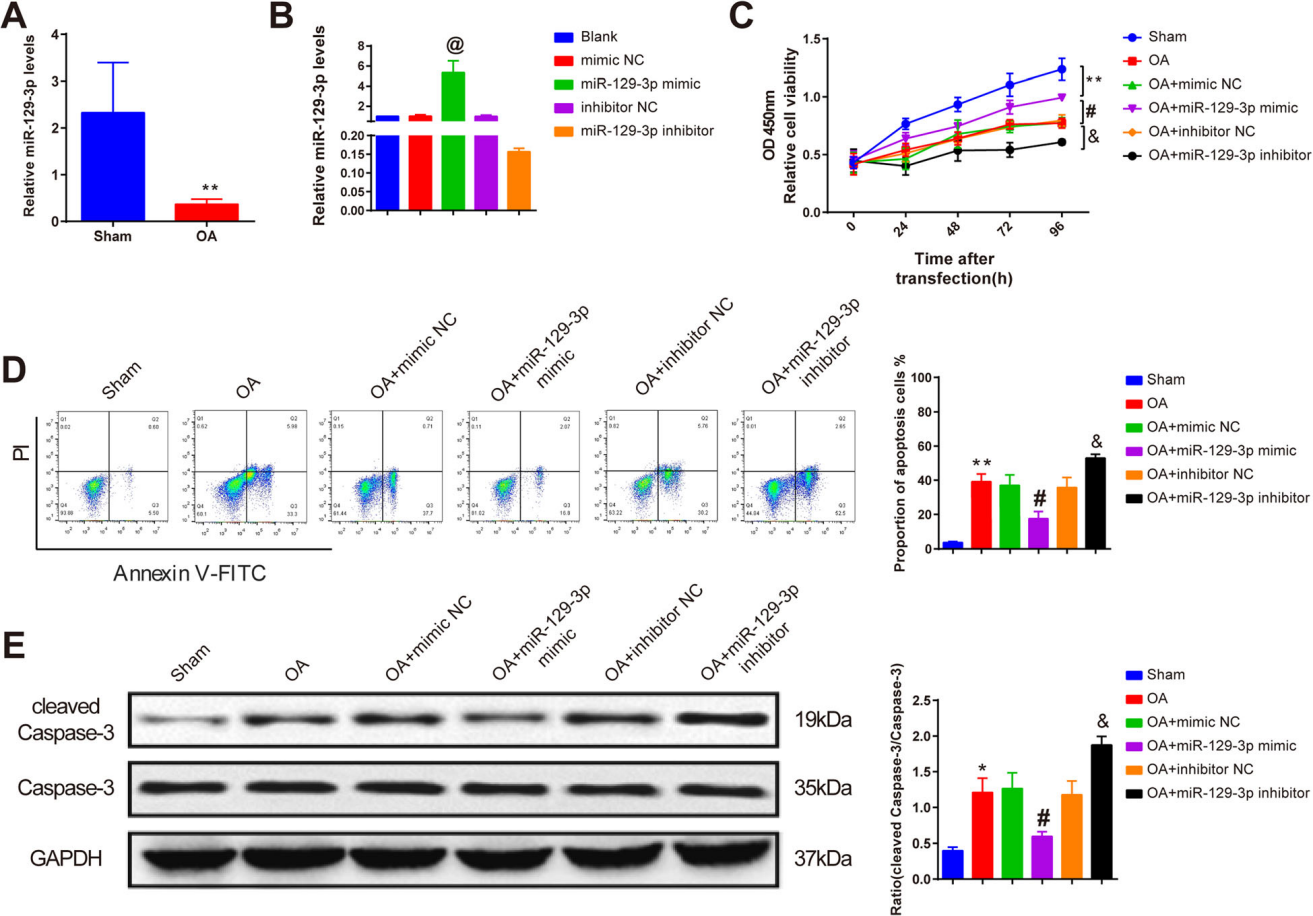
miR-129-3p enhances chondrocyte viability and impairs apoptosis. miR-129-3p expression after OA treatment (**a**) and transfection of miR-129-3p mimic or inhibitor (**b**) as measured by qRT-PCR analysis; the role of miR-129-3p in chondrocyte viability was assessed by CCK8 (**c**); the effect of miR-129-3p on chondrocyte apoptosis and cleaved caspase-3 expression after OA treatment was detected by flow cytometry (**d**) and Western blot (**e**), separately. *P < 0.05, **P < 0.01 compared to the sham group; @ *P* < 0.05 compared to the mimic NC group; #*P* < 0.05 compared to the OA + mimic NC group; &*P* < 0.05 compared to the OA + inhibitor NC group. OA, osteoarthritis

qRT-PCR detected that miR-129-3p expression was declined in chondrocytes after OA cellular model was established compared with the sham group (Fig. 2a, *P* < 0.05). miR-129-3p mimic and miR-129-3p inhibitor were transfected into the chondrocytes, and then, the expression of miR129-3p was measured by qRT-PCR. The results manifested that higher expression of miR-129-3p was found in miR-129-3p mimic-treated chondrocytes, and lower expression was found in miR-129-3p inhibitor-treated chondrocytes (Fig. 2b, *P* < 0.05), indicating significant transfections of miR-129-3p mimic and miR-129-3p inhibitor.

CCK8 assay detected the effect of miR-129-3p on chondrocyte viability. The results demonstrated that OA-treated chondrocytes had lower viability compared with the sham group (*P* < 0.01), while no difference in chondrocyte viability was found in the OA + mimic NC and the OA + inhibitor NC groups compared with OA group (*P* > 0.05) (Fig. 2c). Compared with the OA + mimic NC group, chondrocytes transfected with miR-129-3p mimic had higher viability after OA treatment, and lower viability was shown in the chondrocytes which were transfected with miR-129-3p inhibitor before OA treatment compared with the OA + inhibitor NC group (Fig. 2c, *P* < 0.05).

Then, the role of miR-129-3p in OA-treated chondrocyte apoptosis was measured by flow cytometry. Compared with the sham group, increased apoptosis rate was found in the OA group (*P* < 0.01), but no significant change in apoptosis rate was manifested in mimic NC- and inhibitor NC-treated chondrocytes compared with the OA group (*P* > 0.05) (Fig. 2d). Declined apoptosis rate was found in the OA + miR-129-3p mimic group compared to the OA + mimic NC group, and elevated apoptosis rate was revealed in chondrocytes that were transfected with miR-129-3p inhibitor prior to OA treatment compared with the OA + inhibitor NC group (Fig. 2d, *P* < 0.05).

Consistently, Western blot analysis suggested that OA treatment increased cleaved caspase-3 expression in chondrocytes compared with those in sham-operated chondrocytes (*P* < 0.05), and no marked difference was observed in cleaved caspase-3 expression in the OA + mimic NC and the OA + inhibitor NC group compared with the OA group (*P* > 0.05) (Fig. 2e). While transfection of miR-129-3p mimic before OA treatment downregulated the expression of cleaved caspase-3 compared with the OA + mimic NC group, and the reverse expression pattern was shown in the OA + miR-129-3p inhibitor group, compared to the OA + inhibitor NC group (Fig. 2e, *P* < 0.05). These results confirmed that miR-129-3p could improve chondrocyte viability and hamper OA-induced apoptosis.

### CPEB1 deteriorates the progression of OA by inhibiting chondrocyte viability and enhancing apoptosis

**Fig. 3:**
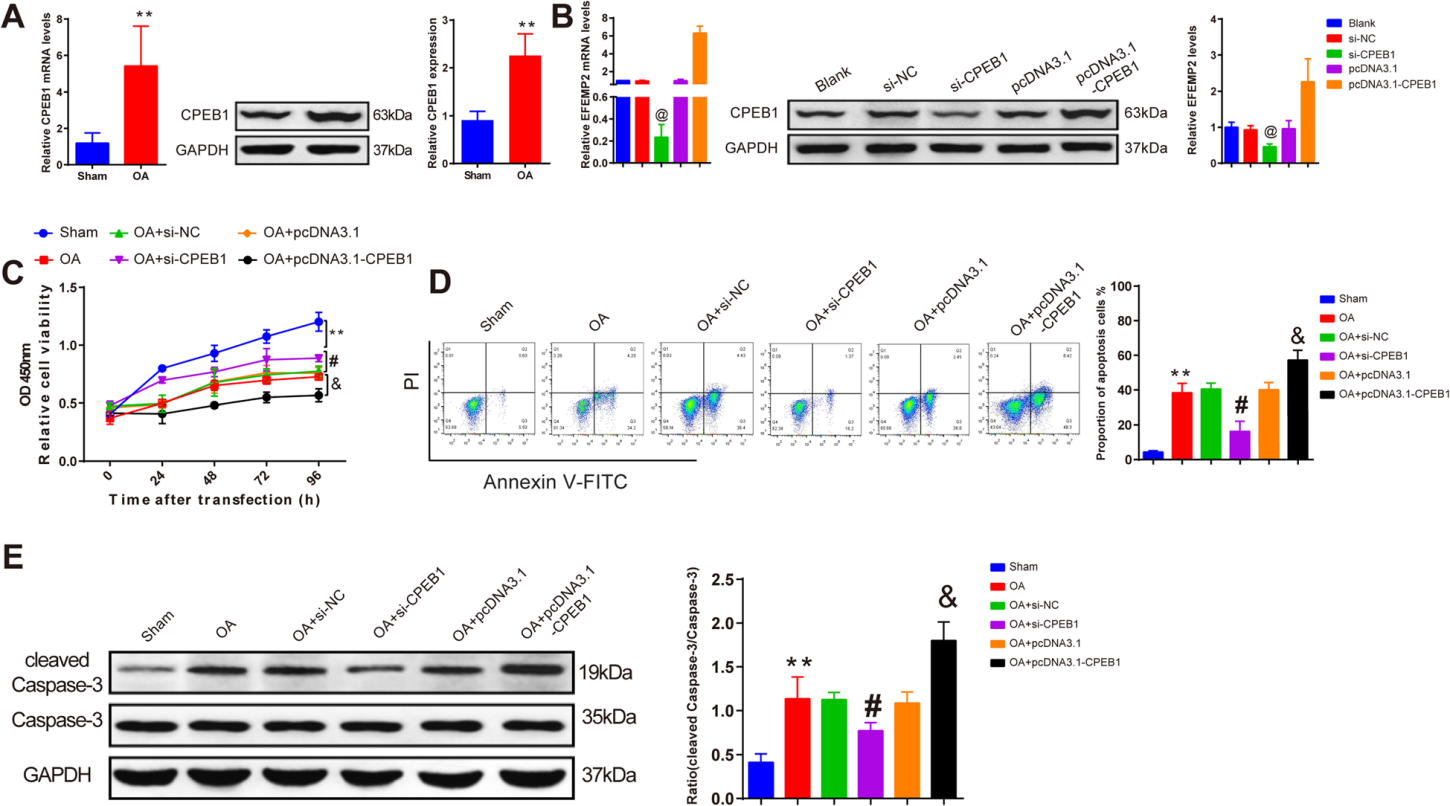
CPEB1 regulates chondrocyte viability and apoptosis. After OA modeling, chondrocytes were transfected with si-CPEB1 or pcDNA3.1-CPEB1; mRNA and protein expression levels of CPEB1 were detected by qRT-PCR and Western blot (**a**, **b**); the role of CPEB1 in chondrocyte viability (**c**), apoptosis (**d**), and cleaved caspase-3 expression (**e**) were measured. *P < 0.05, **P < 0.01 compared to the sham group; @ *P* < 0.05 compared to the si-NC group; #*P* < 0.05 compared to the OA + si-NC group; &*P* < 0.05 compared to the OA + pcDNA3.1 group. OA, osteoarthritis

According to qRT-PCR and Western blot analyses, the mRNA and protein expression levels of CPEB1 were enhanced in chondrocytes after OA treatment compared with those in sham-operated chondrocytes (Fig. 3a, *P* < 0.05). Also, transfection efficiency of si-CPEB1 and pcDNA3.1-CPEB1 in chondrocytes was detected. Chondrocytes had impeded mRNA and protein expression levels of CPEB1 after being transfected with si-CPEB1 compared with the si-NC group, and higher mRNA and protein expression levels of CPEB1 were found in the pcDNA3.1-CPEB1 group than those in the pcDNA3.1 group (Fig. 3b, *P* < 0.05), suggesting expected transfection efficiency in the si-CPEB1 and the pcDNA3.1-CPEB1 groups.

Compared with the OA group, transfections of si-NC and pcDNA3.1 had no significant effect on chondrocyte viability, apoptosis, and cleaved caspase-3 expression (Fig. 3c–e, *P* > 0.05). si-CPEB1-treated chondrocytes had increased cell viability and decreased apoptosis and cleaved caspase-3 expression compared with the OA + si-NC group (Fig. 3c–e, *P* < 0.05). In line with the aforementioned results, reduced cell viability and facilitated apoptosis rate and cleaved caspase-3 expression were found in the OA + pcDNA3.1-CPEB1 group compared with the OA + pcDNA3.1 group (Fig. 3c–e, *P* < 0.05). These results demonstrated that CPEB1 could deteriorate OA progression by inhibiting chondrocyte viability and promoting chondrocyte apoptosis.

### miR-129-3p negatively targets CPEB1

**Fig. 4:**
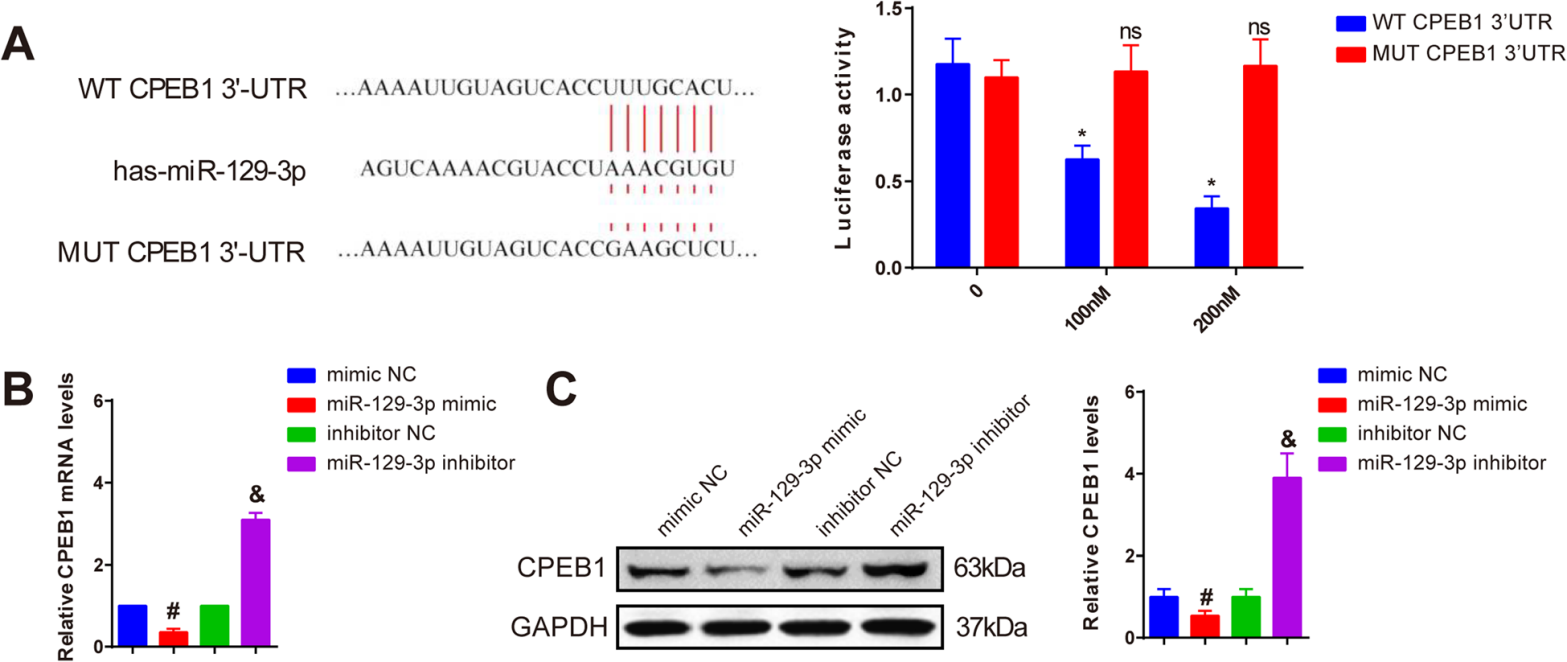
miR-129-3p negatively regulates CPEB1. The binding sites and mutant sites between CPEB1 and miR-129-3p (left), and interactions between miR-129-3p and CPEB1 were detected by dual luciferase reporter assay (right) (**a**); the regulation of miR-129-3p on the mRNA and protein expression levels of CPEB1 as measured by qRT-PCR (**b**) and Western blot (**c**). ns, *P* > 0.05, *P < 0.05 compared to the CPEB1 3′UTR WT group; #*P* < 0.05 compared to the mimic NC group; &*P* < 0.05 compared to inhibitor NC group

In our previous results, miR-129-3p expression was negatively associated with CPEB1 in both cartilage from OA patients and OA-treated chondrocytes. In contrast to CPEB1, miR-129-3p can inhibit cell apoptosis and promote cell viability of chondrocyte. Accordingly, we speculated that miR-129-3p may regulate CPEB1 in OA. TargetScan predicted that there were potential binding sites between miR-129-3p and CPEB1. Luciferase reporter assay revealed that insertion of MUT CPEB1 3′-UTR did not affect the luciferase activity among the groups treated with different concentrations of miR-129-3p mimic, and WT CPEB1 3′-UTR gradually decreased luciferase activity as the increase in miR-129-3p mimic concentration (Fig. 4a, *P* < 0.05), indicating miR-129-3p could bind to CPEB1. qRT-PCR and Western blot further assayed that miR-129-3p targeted CPEB1, evidenced by the decreased expression of CPEB1 in the miR-129-3p mimic group compared with the mimic NC group, and the increased expression of CPEB1 in the miR-129-3p inhibitor group compared with the inhibitor NC group (Fig. 4b, c, *P* < 0.05). Together, miR-129-3p could negatively target CPEB1.

### miR-129-3p inhibits chondrocyte apoptosis through downregulating CPEB1

**Fig. 5:**
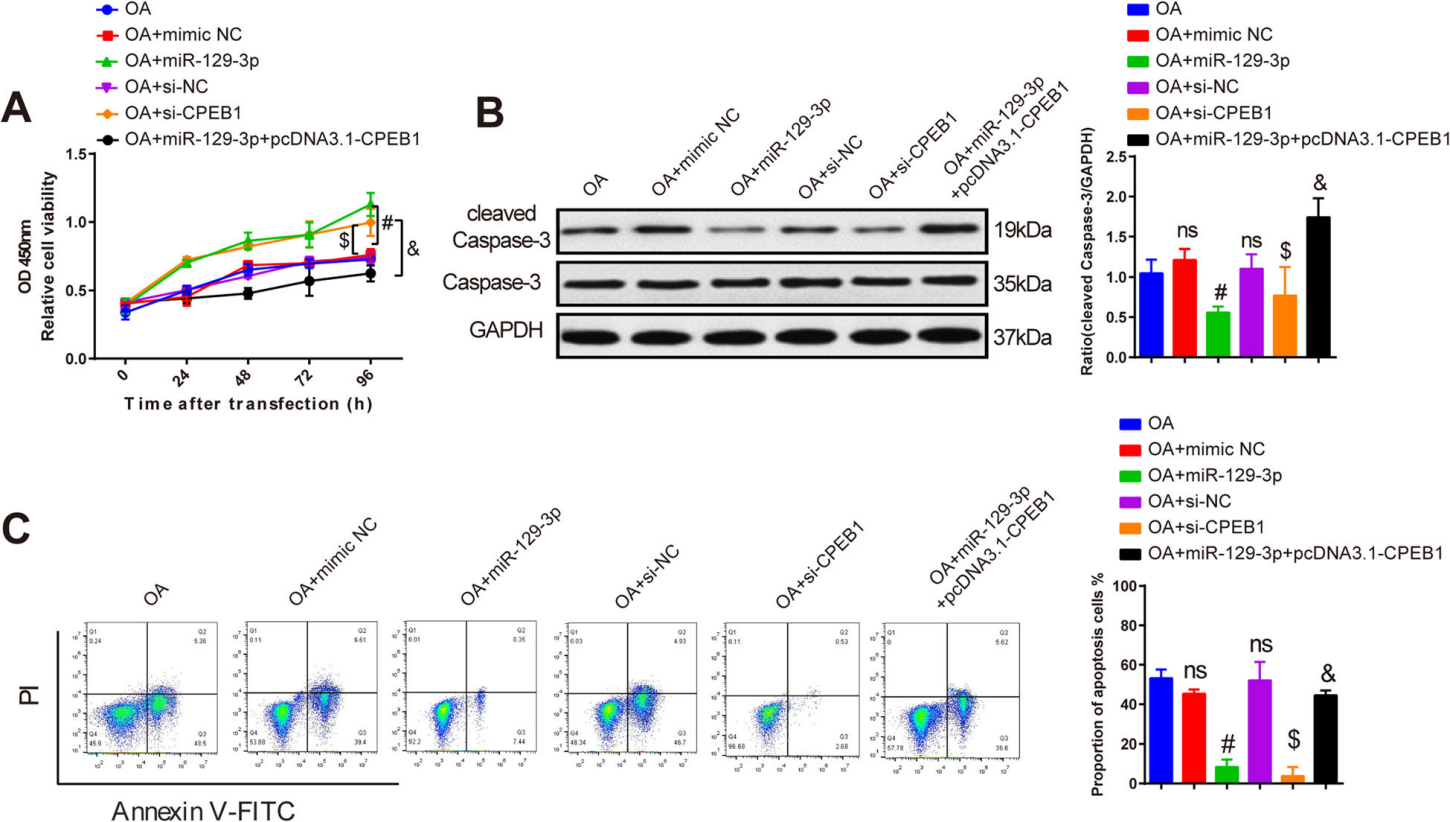
miR-129-3p inhibits chondrocyte apoptosis through regulating CPEB1. CCK8 detected chondrocyte viability in each group (**a**); detection of cleaved caspase-3 expression by Western blot (**b**); apoptosis rate was examined by flow cytometry (**c**). ns, *P* > 0.05 compared to the OA group; #*P* < 0.05 compared to the OA + mimic NC group; $*P* < 0.05 compared to the OA + si-NC group; &*P* < 0.05 compared to the OA + miR-129-3p group. OA, osteoarthritis

It had been confirmed that miR-129-3p could negatively regulate CPEB1 and inhibit chondrocyte apoptosis; thereby, we explored whether miR-129-3p regulated CPEB1 to implicate in chondrocyte apoptosis. CCK8 assay denoted that compared to the OA group, separately transfection of mimic NC or pcDNA3.1 had no notable effects on chondrocyte viability, apoptosis and cleaved caspase-3 expression, and transfection of miR-129-3p mimic or si-CPEB1enhanced chondrocyte viability and impeded chondrocyte apoptosis and cleaved caspase-3 expression compared with the OA + mimic NC or the OA + si-NC group (Fig. 5a–c, *P* < 0.05), while co-transfection of miR-129-3p mimic and pcDNA3.1-CPEB1 reduced cell viability and exacerbated chondrocyte apoptosis and cleaved caspase-3 expression compared with the OA + miR-129-3p mimic group (Fig. 5a–c, *P* < 0.05). All this suggested that miR-129-3p negatively targeted CPEB1 to enhance chondrocyte viability and inhibit apoptosis.

## Discussion

Accumulating evidence has shown that chondrocyte apoptosis plays a key role in the occurrence and development of OA [16, 17]. Meanwhile, numerous miRs have been reported to implicate in chondrocyte apoptosis in OA [4, 18]. However, the mechanism underlying miR-129-3p in OA progression needs further exploration. In the current study, we investigated the inhibitory effect of miR-129-3p on OA induced by knee joint fracture.

A previous study suggested that miR-129-3p was downregulated in rheumatoid arthritis and inhibition of miR-129-3p expression contributed to IL-17 expression [14]. Herein, miR-129-3p was downregulated in the cartilage from OA patients, indicating the potential implication of miR-129-3p in OA progression. Meanwhile, upregulated CPEB1 expression was found in the cartilage of OA patients. As a highly conserved RNA-binding protein, CPEB1 mediates the extension of the mRNA polyadenylation tail and facilitates the translation of target genes [19]. Notably, a previous study has reported that CPEB1 depletion decreased the production of pro-inflammatory cytokines in mouse embryo fibroblasts [20]. TNF-α, a crucial pro-inflammatory cytokine related to OA, could intervene catabolic and anabolic signals in chondrocytes and implicate in cell apoptosis [21]. It has been mentioned that in fracture-induced OA, TNF-α level was notably upregulated in the cartilage [22]. Accordingly, in this study, TNF-α was used to induce OA in chondrocytes. The chondrocytes treated with TNF-α were transfected with miR-129-3p mimic and miR-129-3p inhibitor to identify the effect of miR-129-3p on chondrocyte apoptosis induced by OA. According to the results, miR-129-3p overexpression rescued chondrocyte viability and impaired apoptosis and cleaved caspase-3 expression, and miR-129-3p inhibition presented the reverse function, unraveling the potential therapeutic effect of miR-129-3p on OA-induced chondrocyte apoptosis. miR-129-3p has been reported to be inhibited in palmitic acid-treated cardiomyocytes, and overexpression of miR-129-3p could ameliorate cardiomyocyte inflammation and apoptosis [13]. Moreover, CPEB1 silence markedly enhanced the cell viability and inhibited chondrocyte apoptosis. Consistently, CPEB1 overexpression hampered chondrocyte viability and promoted OA-treated chondrocyte apoptosis.

The correlation analysis disclosed the negative correlation between miR-129-3p and CPEB1 in the cartilage of OA patients and OA-treated chondrocytes. Hence, we speculated that miR-129-3p regulated CPEB1 to implicate in chondrocyte apoptosis. Dual luciferase reporter assay uncovered that luciferase activity was changed after the insertion of wt-CPEB1 3′UTR. Additionally, lower luciferase activity existed in the group with higher miR-129-3p mimic concentration. More importantly, miR-129-3p overexpression could reduce CPEB1 expression, and miR-129-3p inhibitor could enhance CPEB1 expression. Hence, we confirmed that CPEB1 was a target gene of miR-129-3p, and miR-129-3p could negatively regulate CPEB1 expression. Then, our results further confirmed that both miR-129-3p overexpression and CPEB1 silence could rescue chondrocyte viability and hamper apoptosis, evidenced by decreased cleaved caspase-3 expression. miR-129-3p has been reported to implicate in breast cancer cell apoptosis [12], and CPEB1 inhibition could suppress oxidative stress, inflammation, and apoptosis in oxidized low-density lipoprotein–induced endothelial dysfunction [23]. Moreover, overexpression of CPEB1 could reduce the protective effect of miR-129-3p on chondrocyte viability and apoptosis. Similar findings regarding the role of CPEB1 in OA has been reported. CPEB1 expression was enhanced in AC from patients with posttraumatic ankle OA (PTAOA), and CPEB1 overexpression was proved to aggravate the catabolic effect of IL-1β on chondrocyte in vitro [24]. Besides, CPEB1 has been reported as a target gene of miR and participates in cell viability and apoptosis in endometrial cancer cell [25].

In conclusion, our study showed that miR-129-3p could protect chondrocyte from OA-induced apoptosis, possibly via downregulating CPEB1, strongly evidencing a potential therapeutic strategy of OA progression.

## Supplementary Information

**Additional file 1.**

**Additional file 2.**

**Additional file 3.**

**Additional file 4.**

**Additional file 5.**

**Additional file 6.**

**Additional file 7.**

**Additional file 8.**

**Additional file 9.**

**Additional file 10.**

**Additional file 11.**

**Additional file 12.**

**Additional file 13.**

**Additional file 14.**

**Additional file 15.**

**Additional file 16.**

**Additional file 17.**

**Additional file 18.**

**Additional file 19.**

**Additional file 20.**

## Data Availability

The datasets used or analyzed during the current study are available from the corresponding author on reasonable request.

## Abbreviations

OA: Osteoarthritis
OD: Optical density
