# Supplementary figures and images for: miR-129-3p alleviates chondrocyte apoptosis in knee joint fracture-induced osteoarthritis through CPEB1
Source: J Orthop Surg Res. 2020 Nov 23;15:552. doi: 10.1186/s13018-020-02070-1 (PMC7684967; doi:10.1186/s13018-020-02070-1)

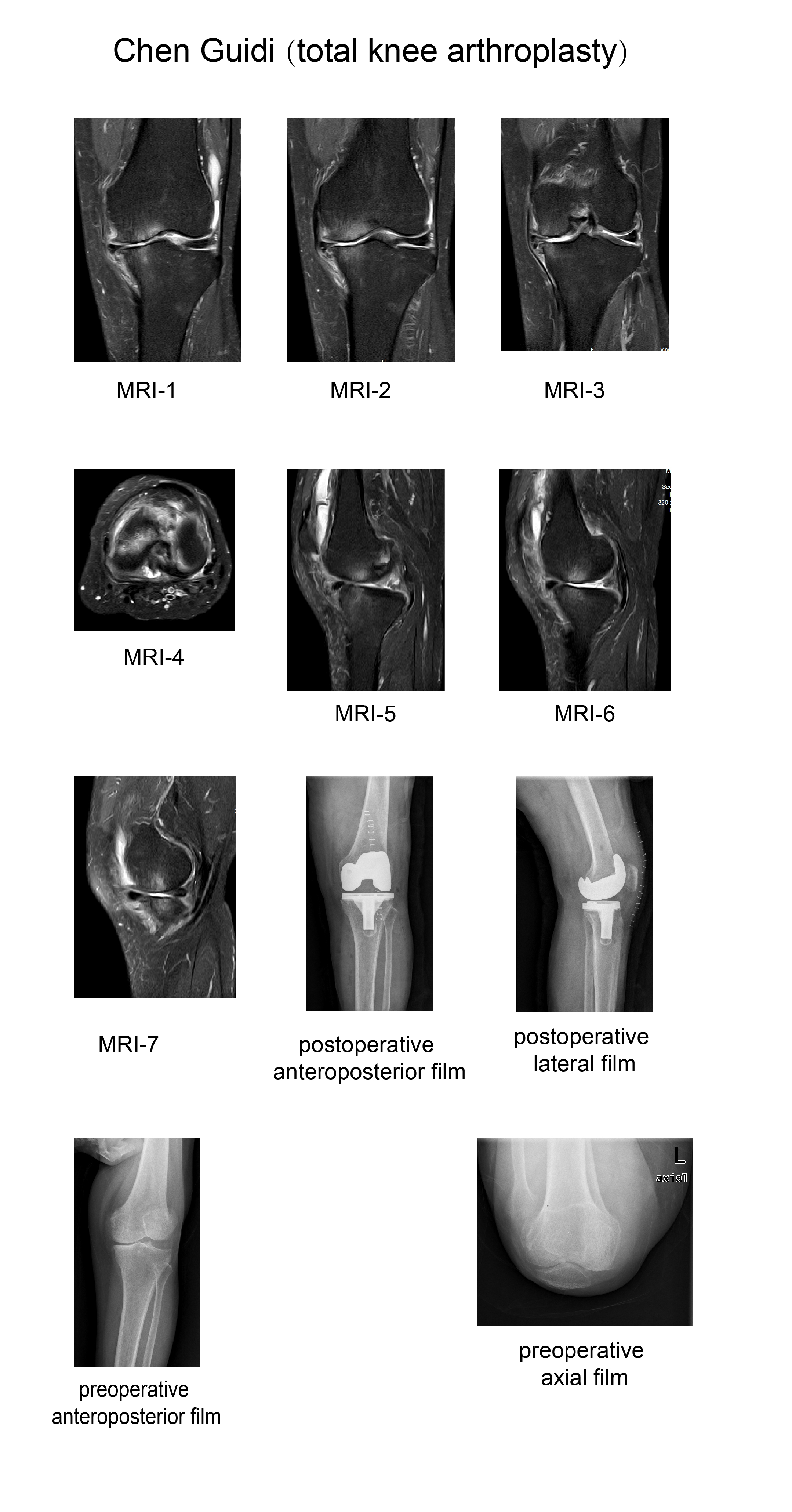

Supplement: Supplementary file 1 — Additional file 1. [file 13018_2020_2070_MOESM1_ESM.jpg]

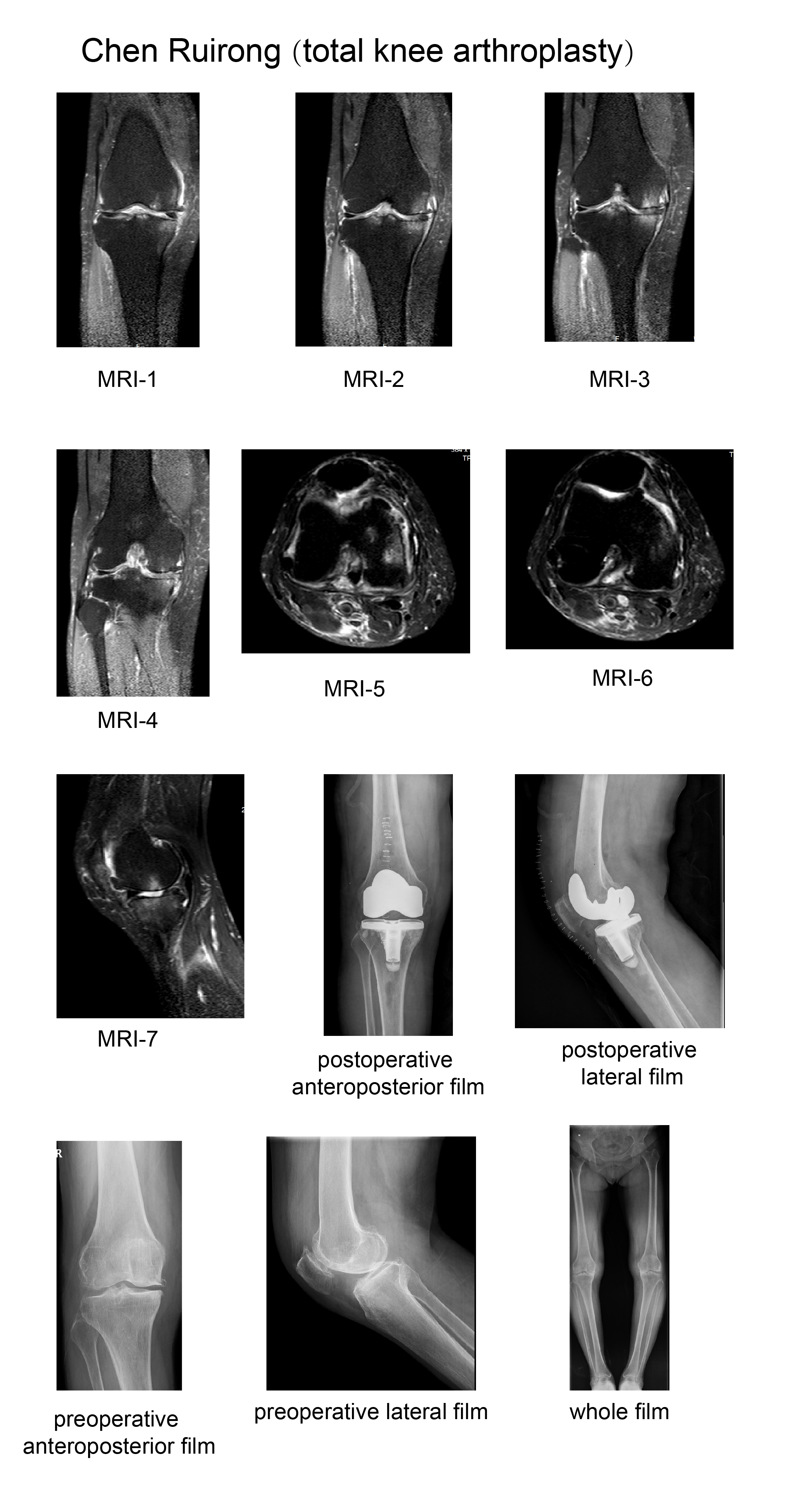

Supplement: Supplementary file 2 — Additional file 2. [file 13018_2020_2070_MOESM2_ESM.jpg]

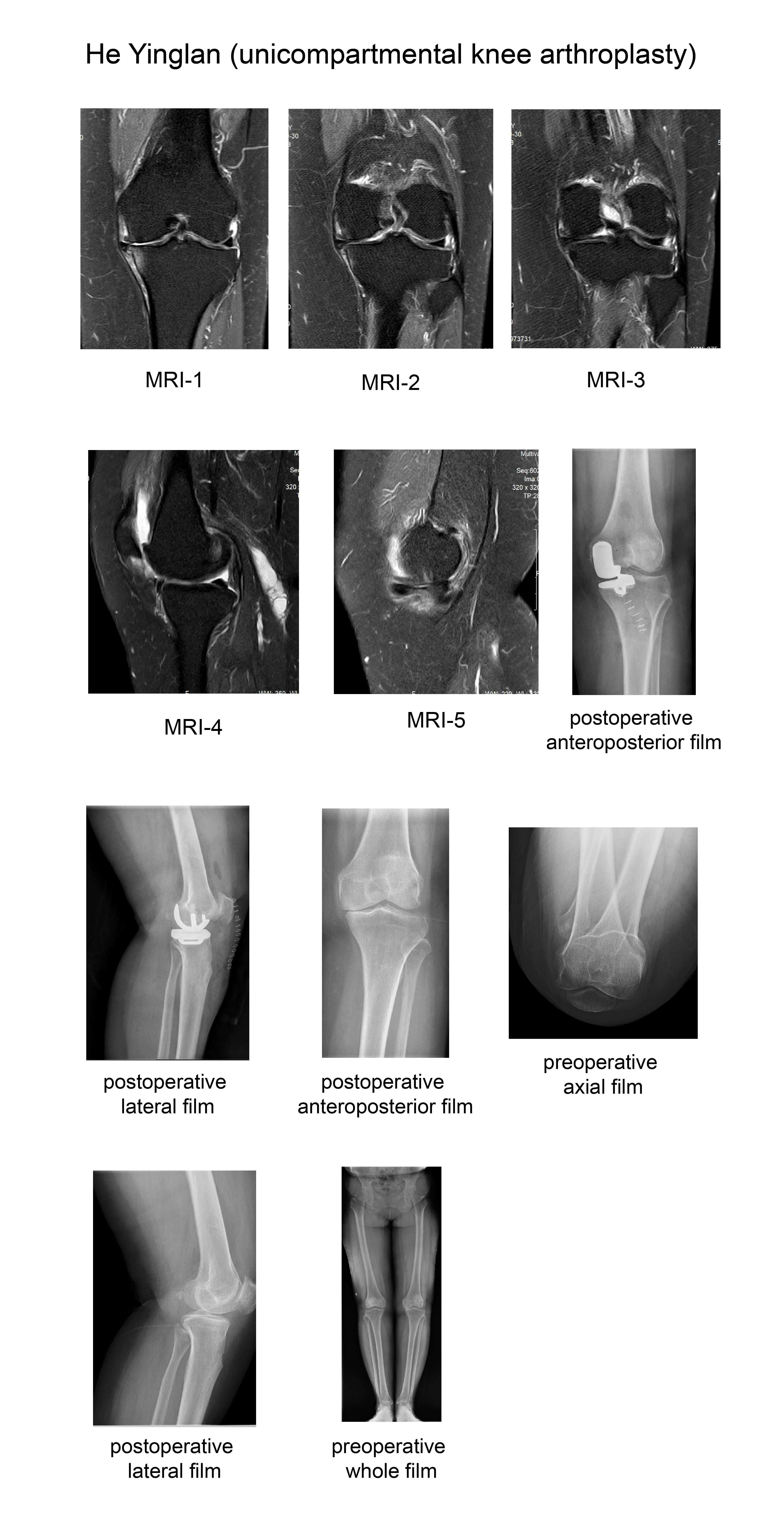

Supplement: Supplementary file 3 — Additional file 3. [file 13018_2020_2070_MOESM3_ESM.jpg]

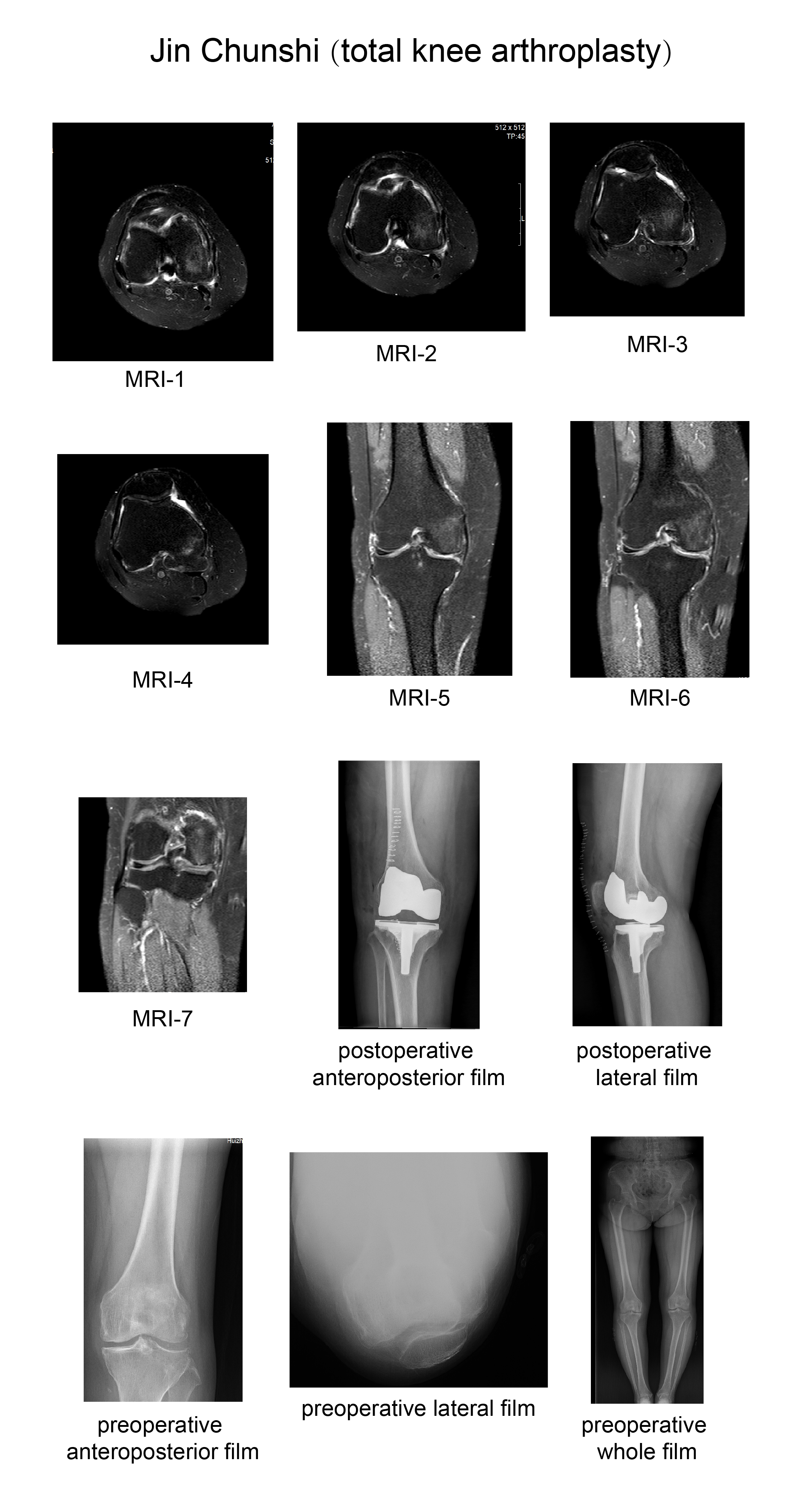

Supplement: Supplementary file 4 — Additional file 4. [file 13018_2020_2070_MOESM4_ESM.jpg]

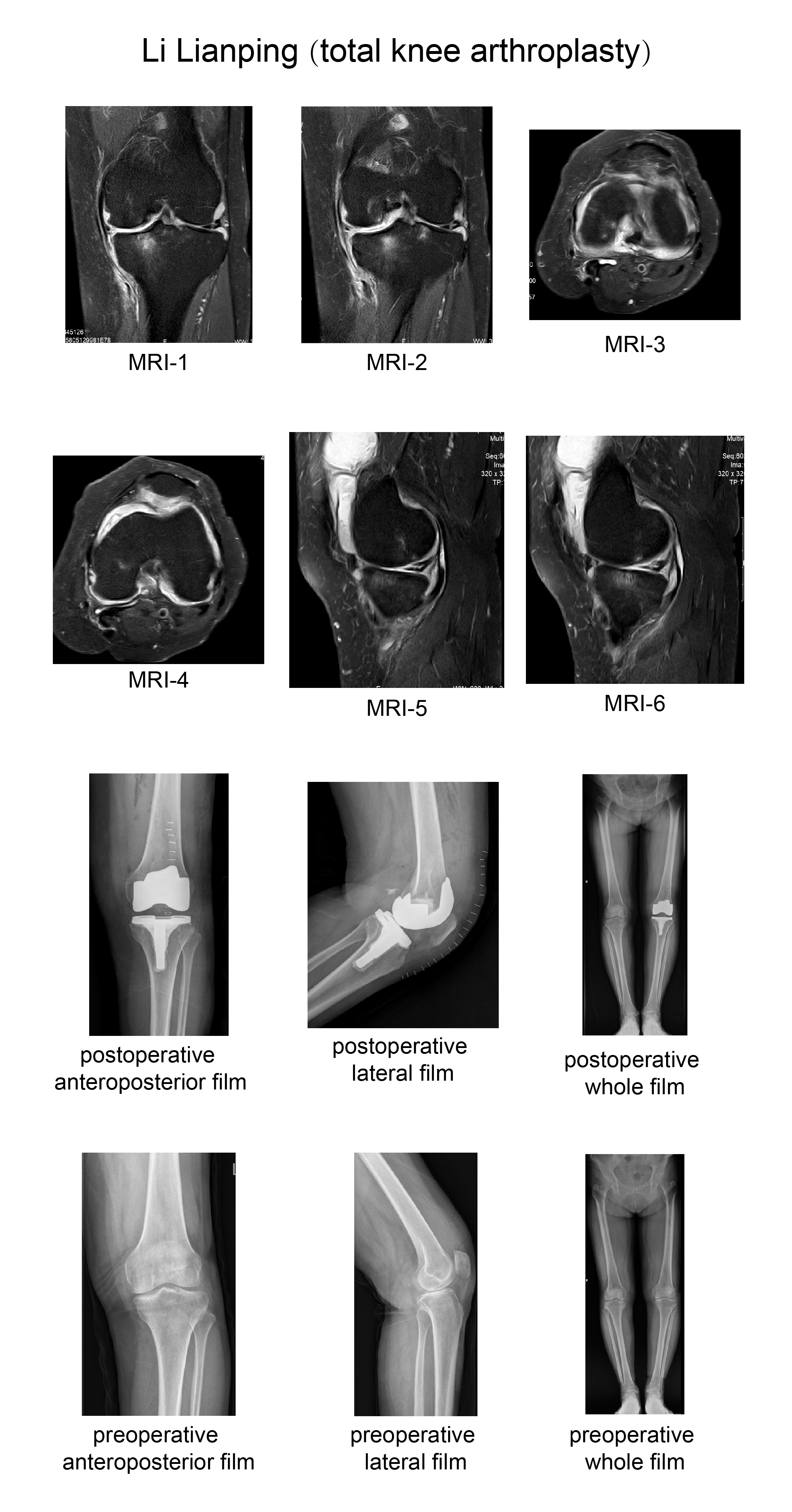

Supplement: Supplementary file 5 — Additional file 5. [file 13018_2020_2070_MOESM5_ESM.jpg]

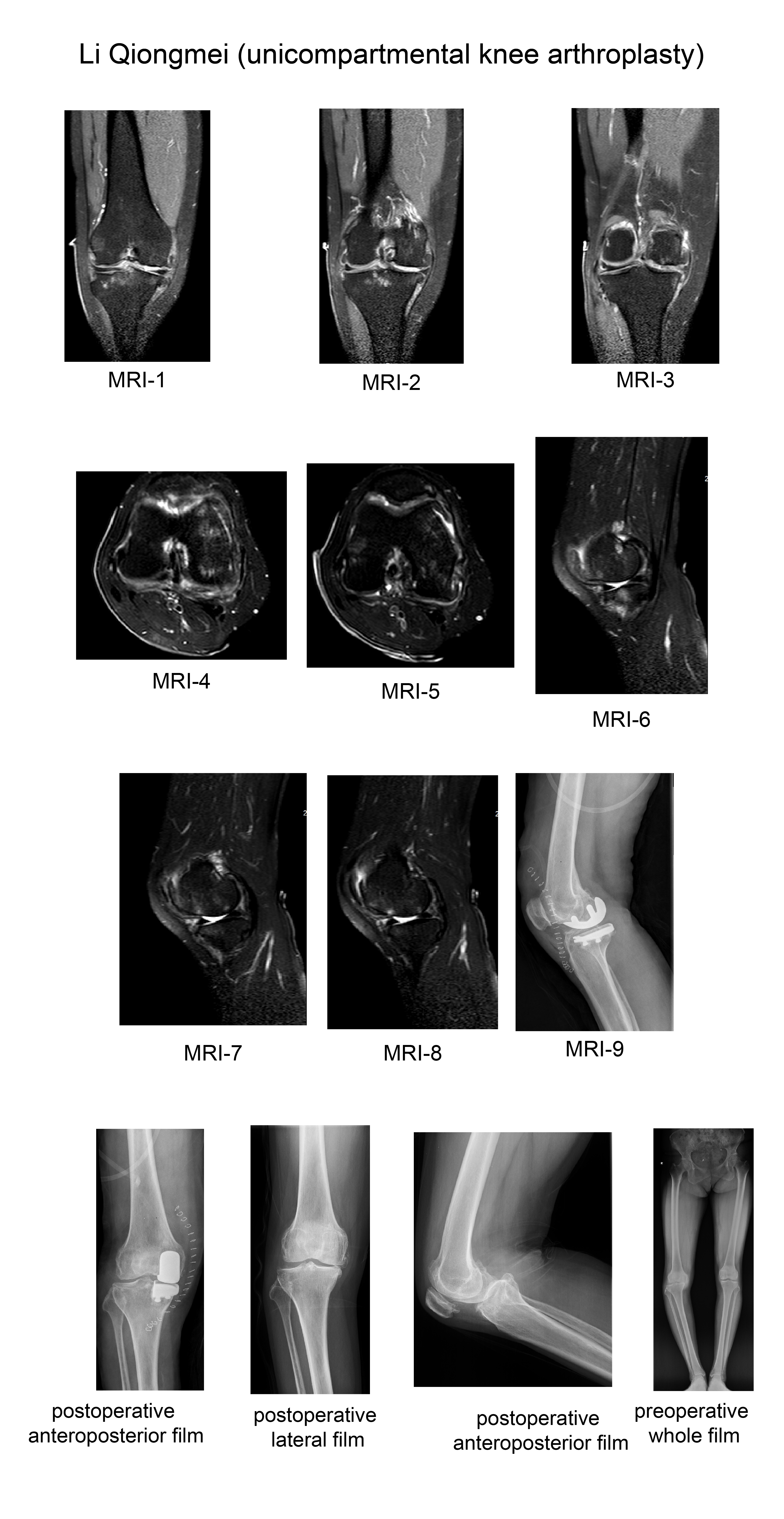

Supplement: Supplementary file 6 — Additional file 6. [file 13018_2020_2070_MOESM6_ESM.jpg]

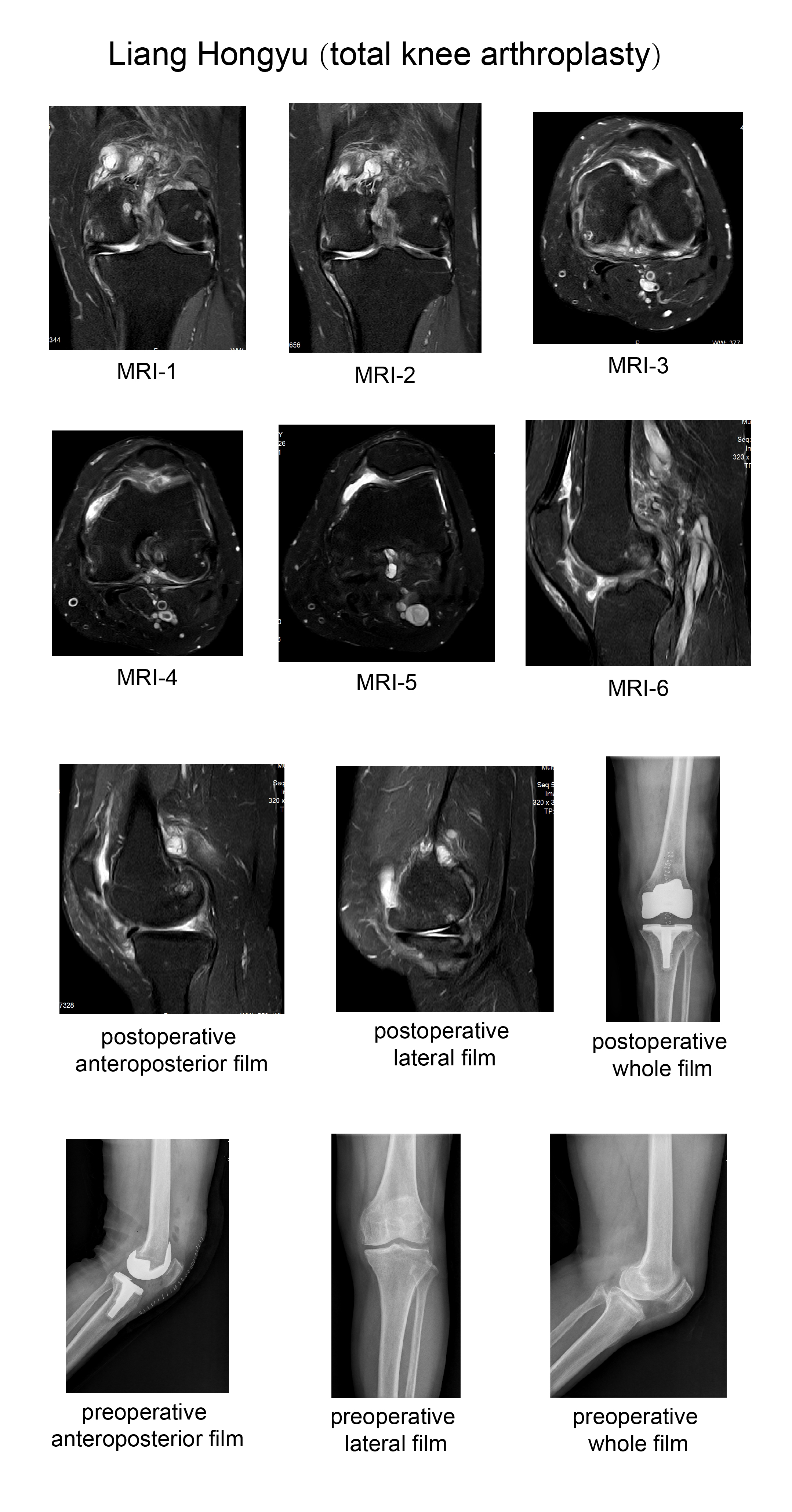

Supplement: Supplementary file 7 — Additional file 7. [file 13018_2020_2070_MOESM7_ESM.jpg]

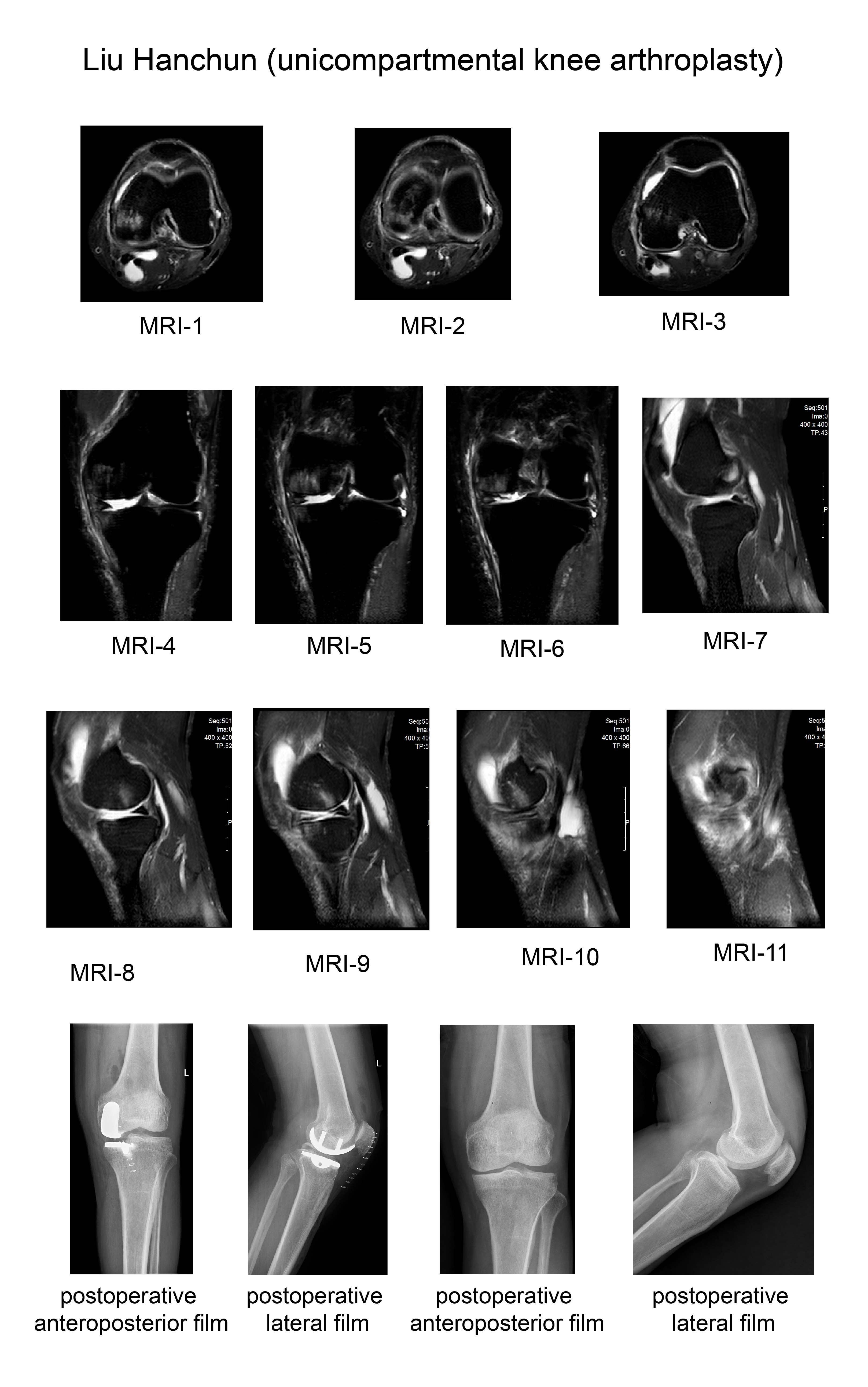

Supplement: Supplementary file 8 — Additional file 8. [file 13018_2020_2070_MOESM8_ESM.jpg]

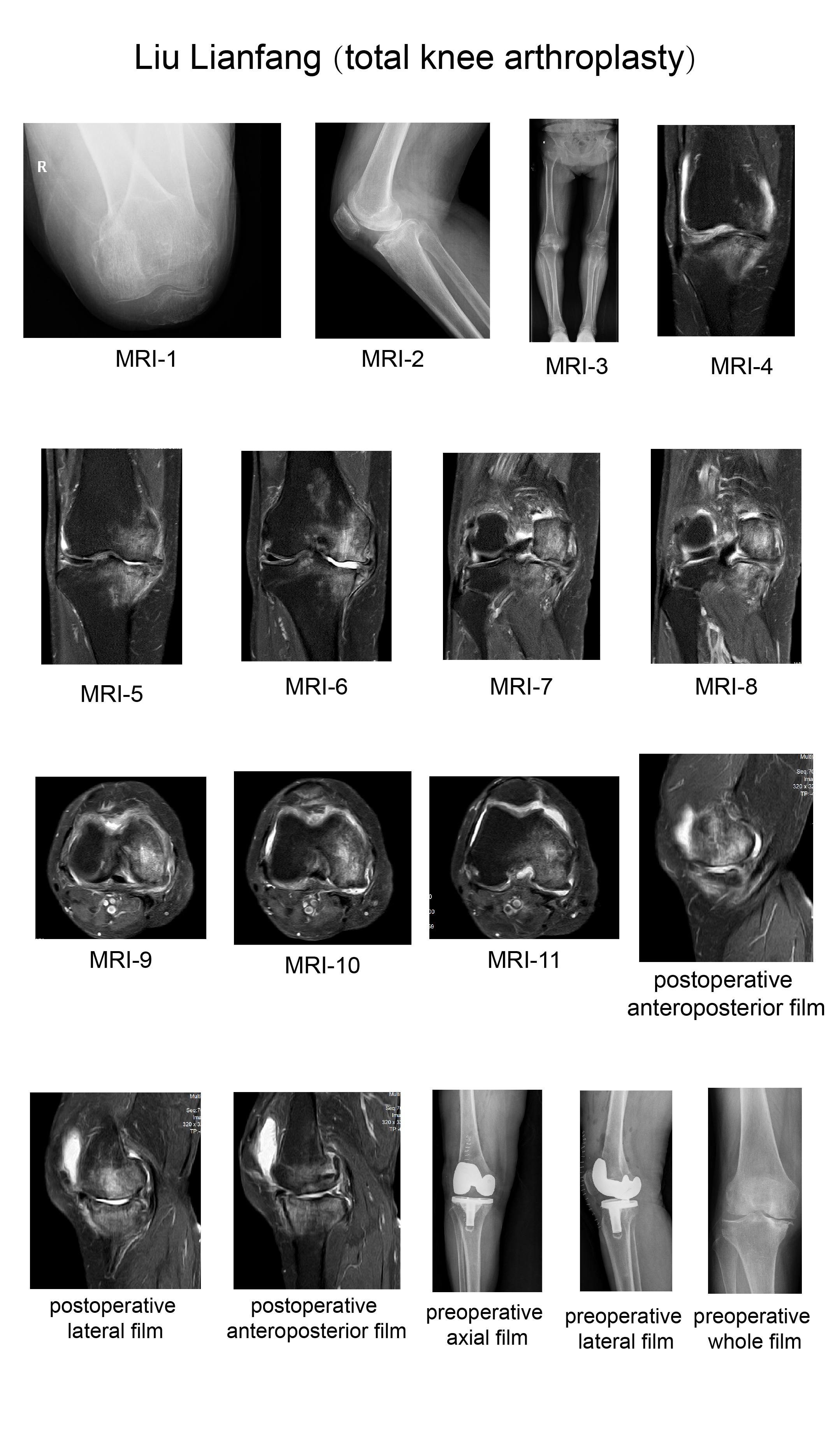

Supplement: Supplementary file 9 — Additional file 9. [file 13018_2020_2070_MOESM9_ESM.jpg]

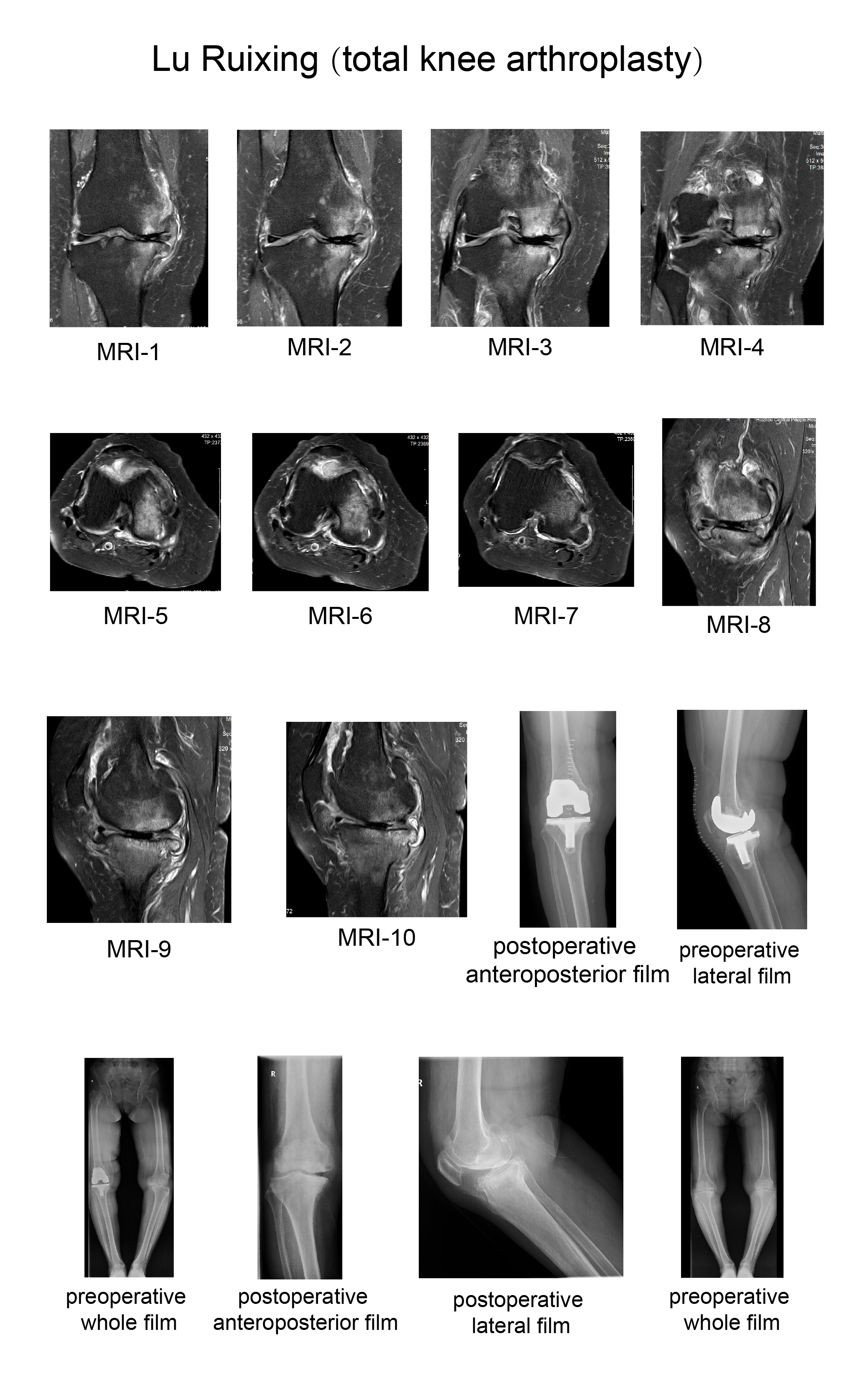

Supplement: Supplementary file 10 — Additional file 10. [file 13018_2020_2070_MOESM10_ESM.jpg]

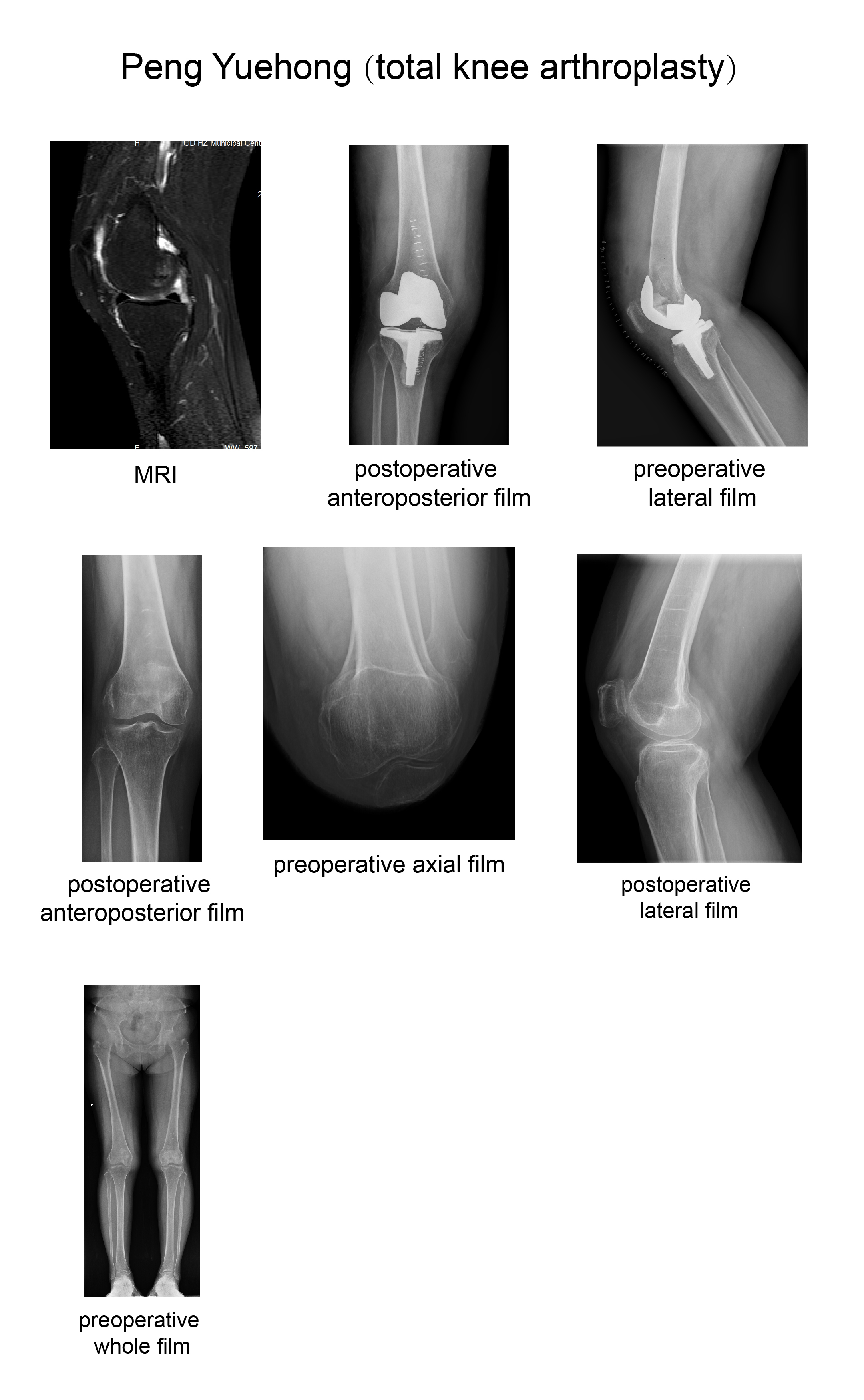

Supplement: Supplementary file 11 — Additional file 11. [file 13018_2020_2070_MOESM11_ESM.jpg]

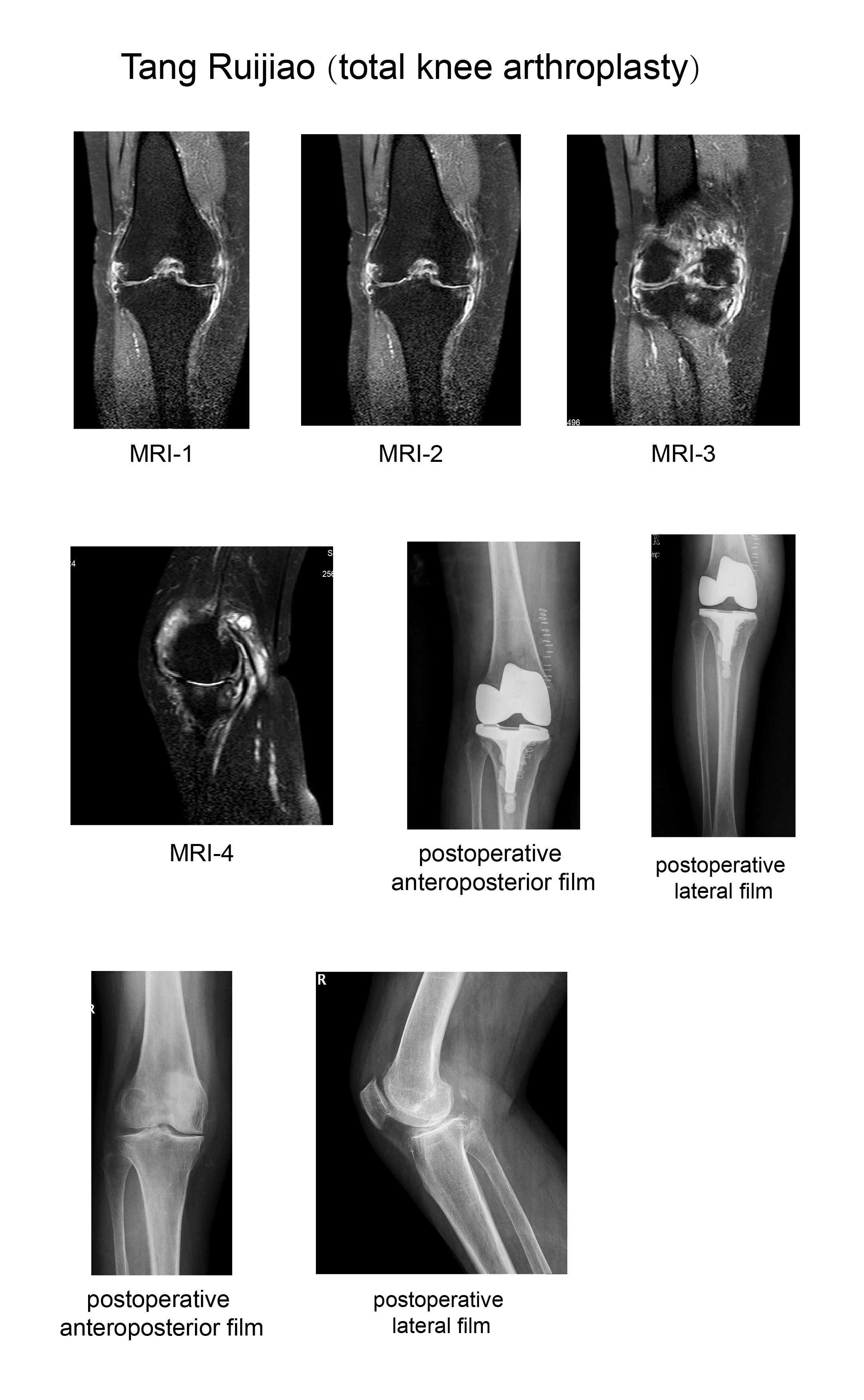

Supplement: Supplementary file 12 — Additional file 12. [file 13018_2020_2070_MOESM12_ESM.jpg]

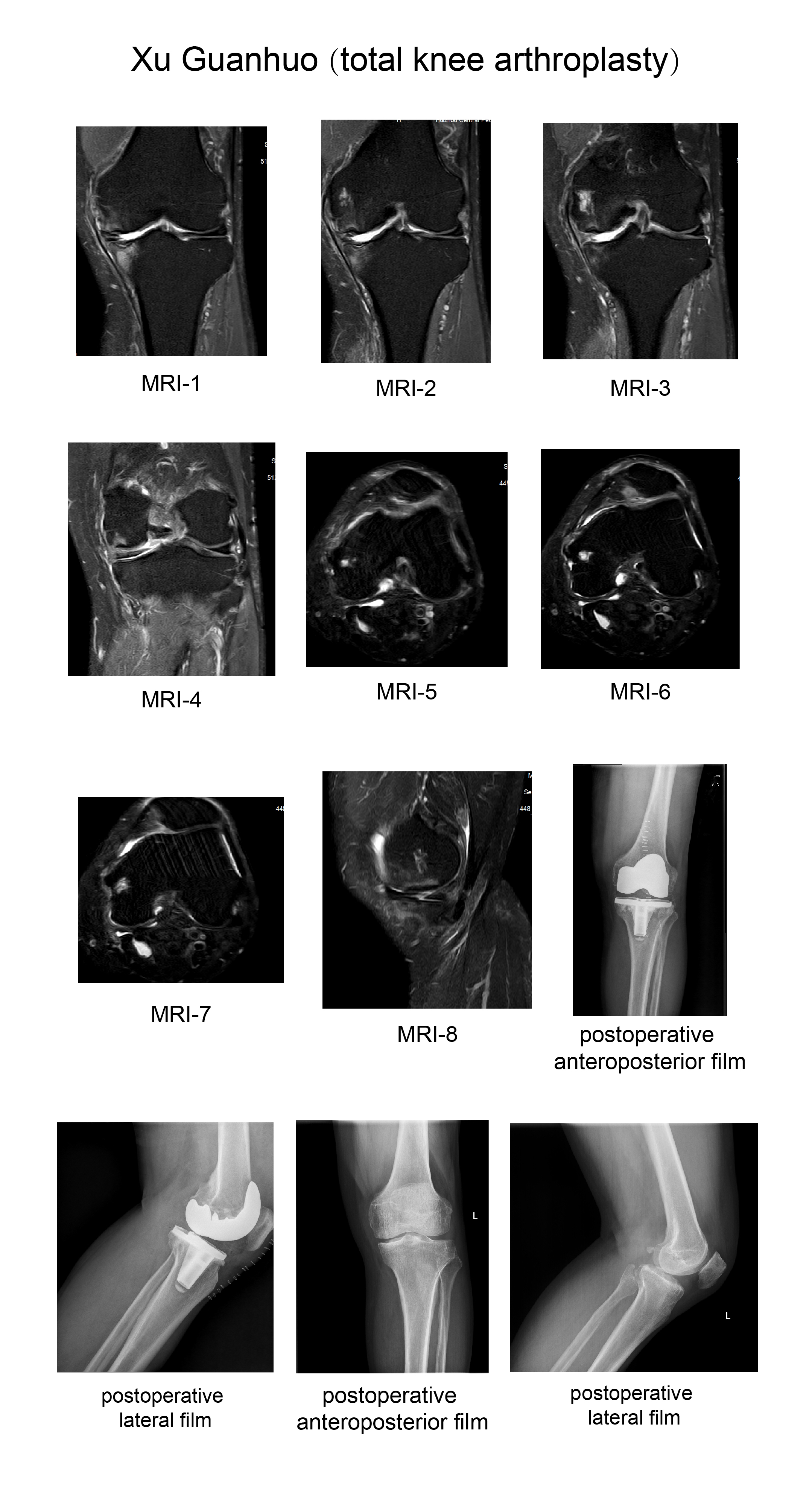

Supplement: Supplementary file 13 — Additional file 13. [file 13018_2020_2070_MOESM13_ESM.jpg]

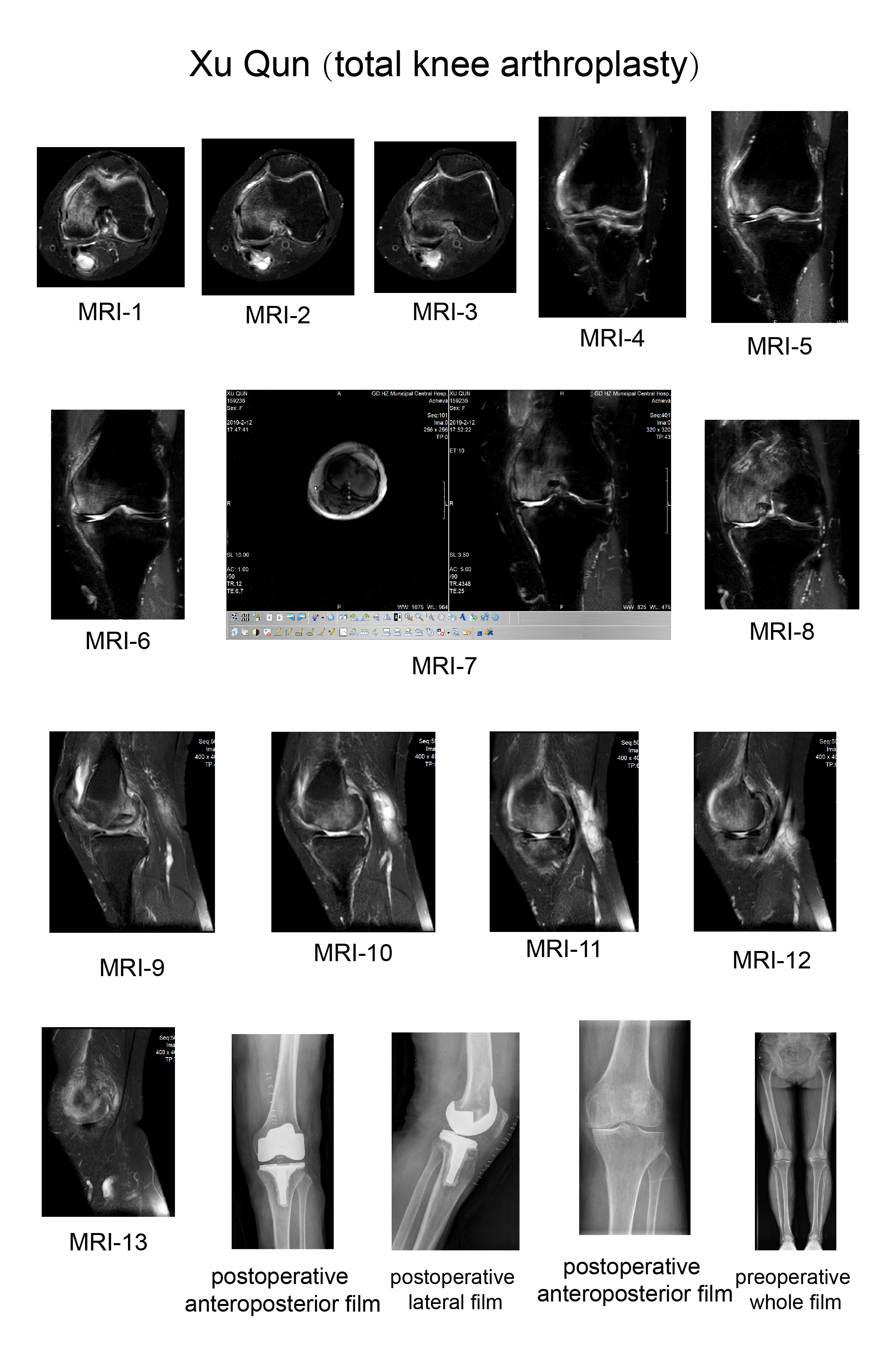

Supplement: Supplementary file 14 — Additional file 14. [file 13018_2020_2070_MOESM14_ESM.jpg]

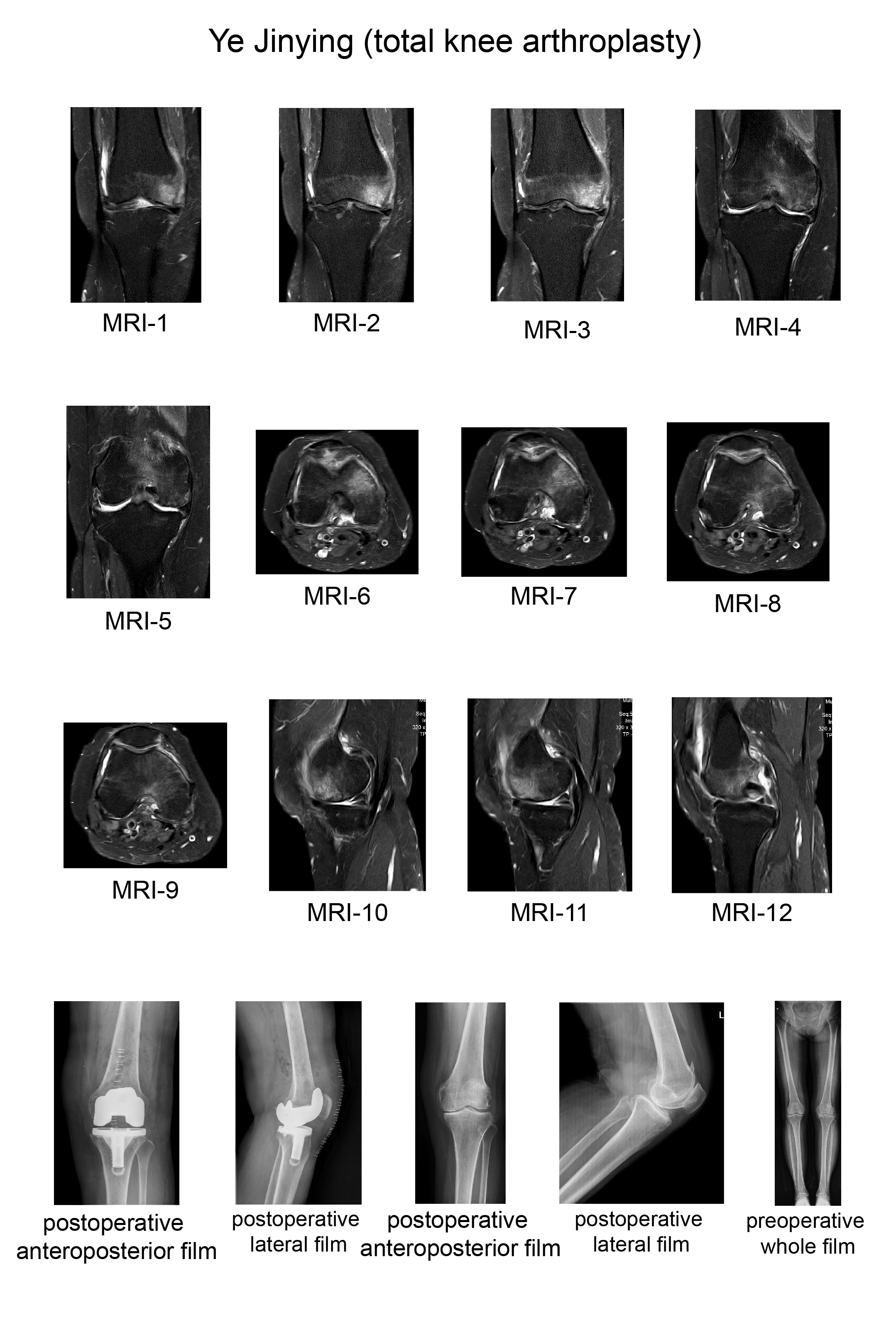

Supplement: Supplementary file 15 — Additional file 15. [file 13018_2020_2070_MOESM15_ESM.jpg]

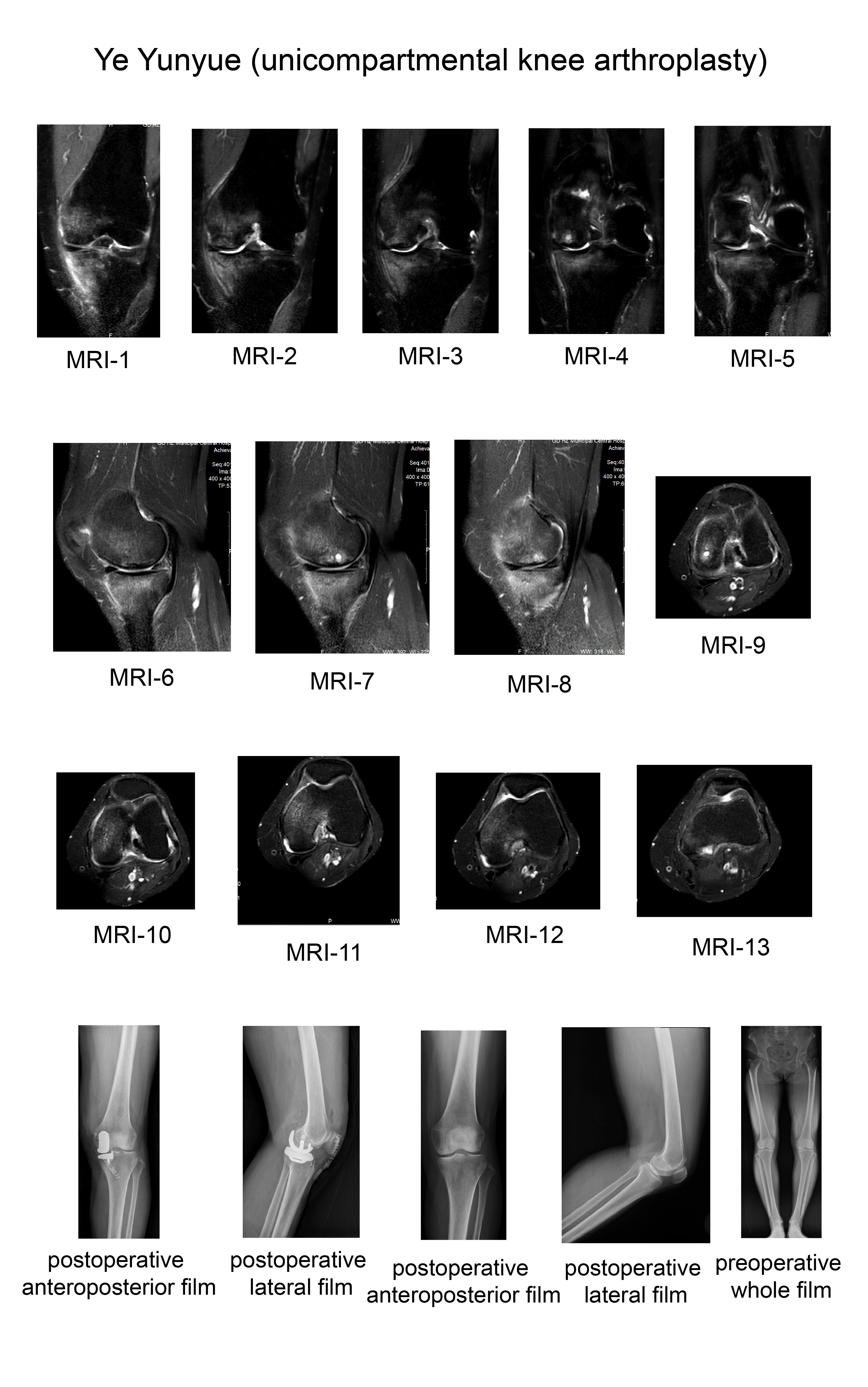

Supplement: Supplementary file 16 — Additional file 16. [file 13018_2020_2070_MOESM16_ESM.jpg]

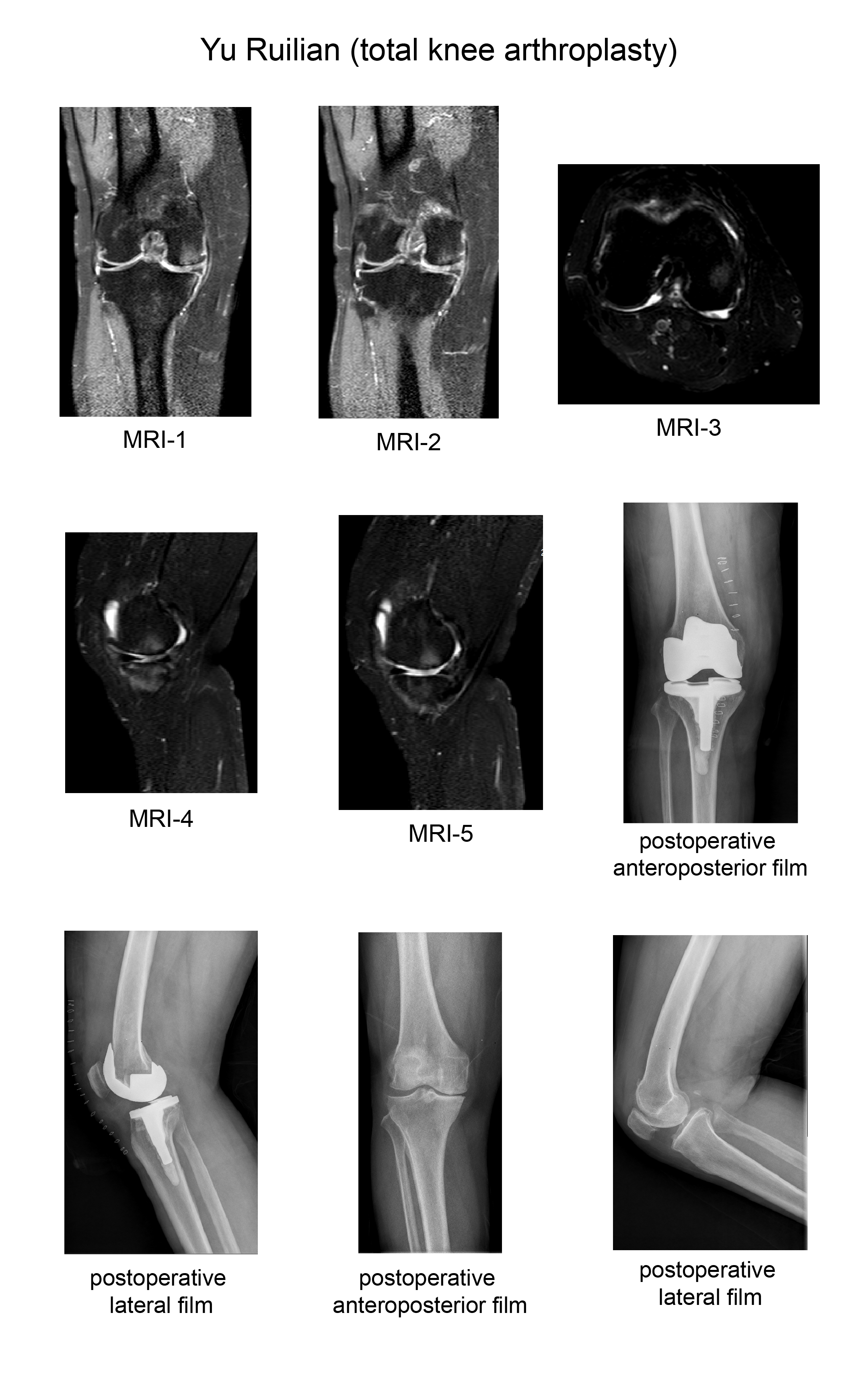

Supplement: Supplementary file 17 — Additional file 17. [file 13018_2020_2070_MOESM17_ESM.jpg]

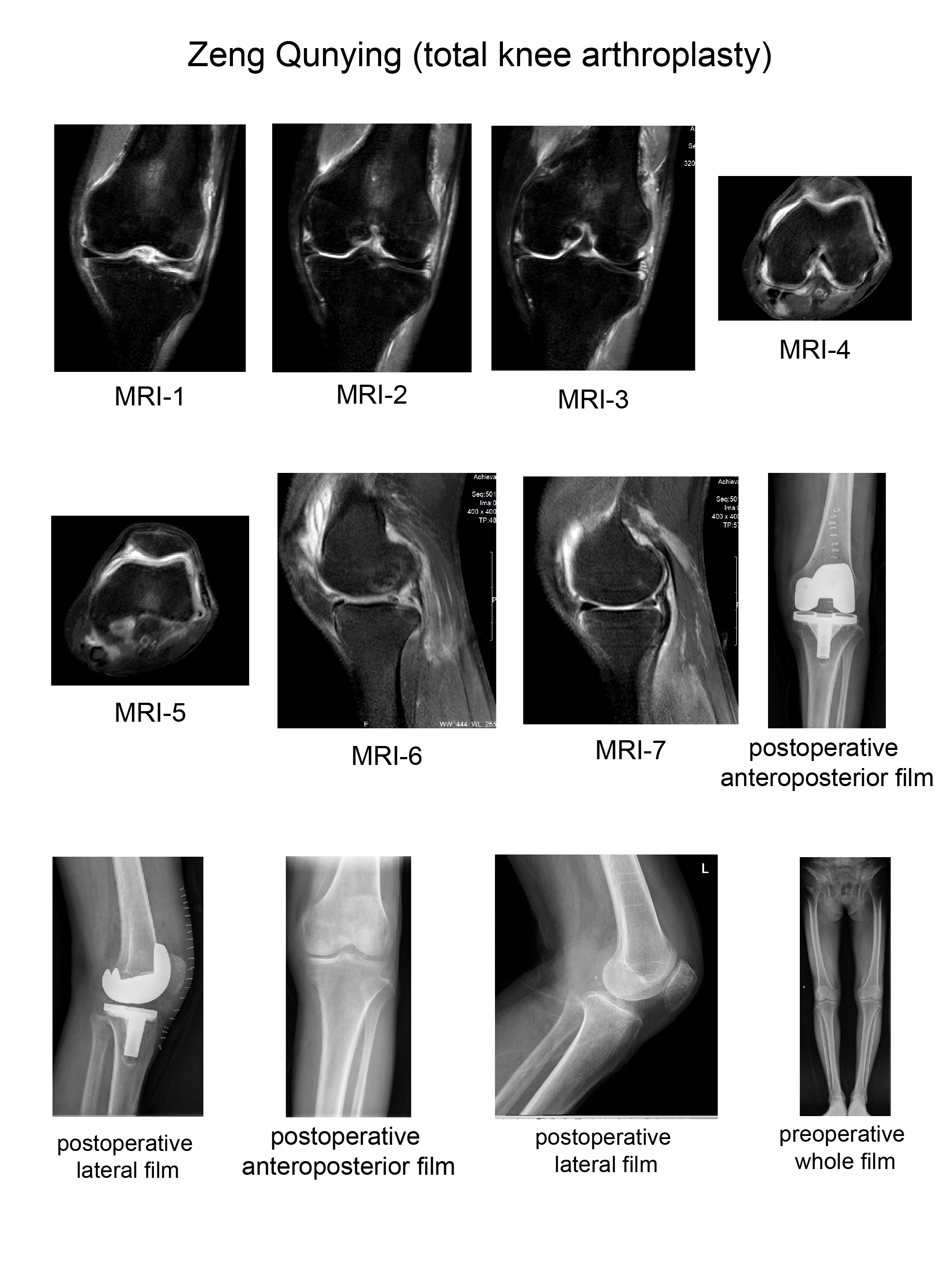

Supplement: Supplementary file 18 — Additional file 18. [file 13018_2020_2070_MOESM18_ESM.jpg]

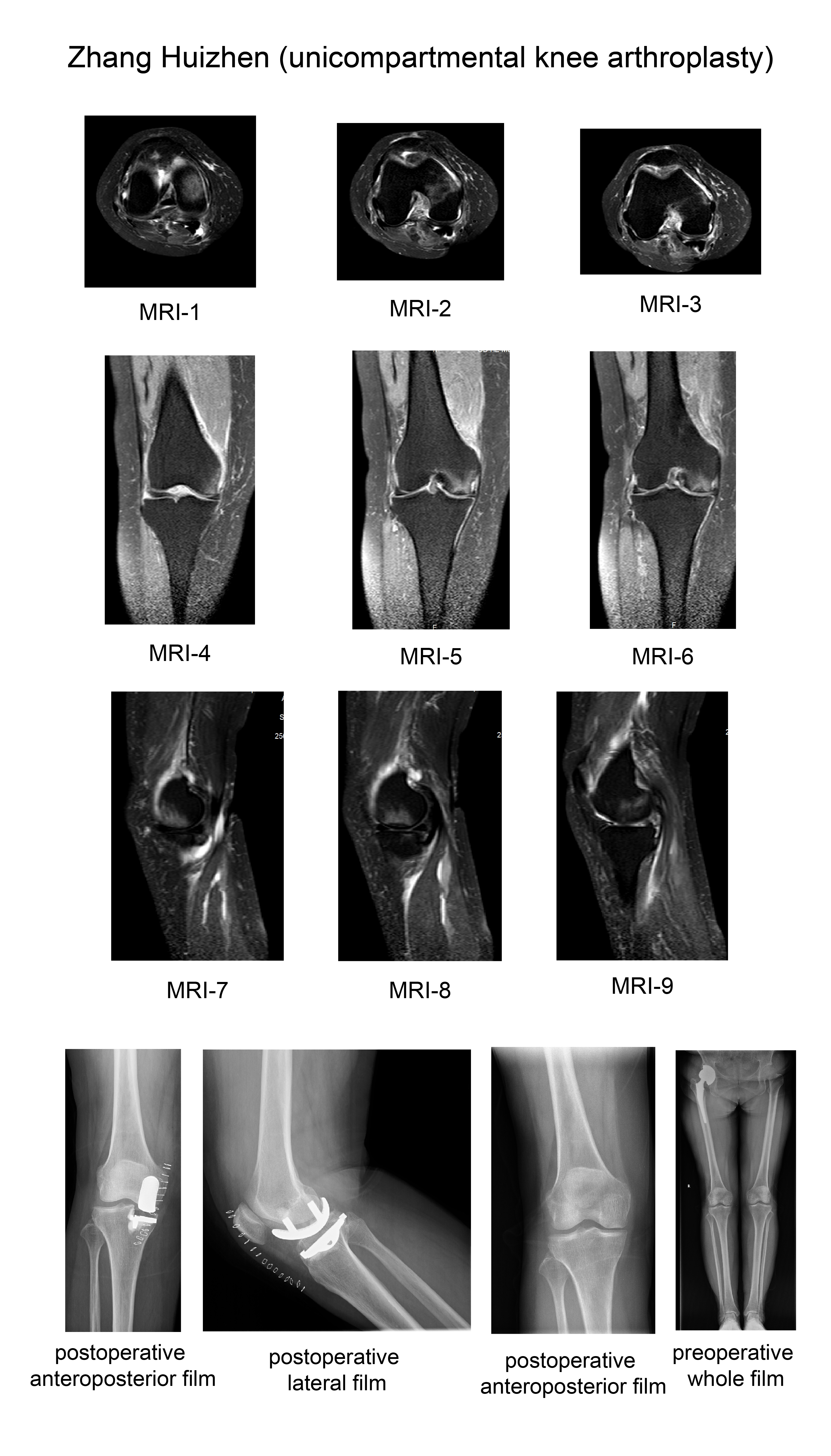

Supplement: Supplementary file 19 — Additional file 19. [file 13018_2020_2070_MOESM19_ESM.jpg]

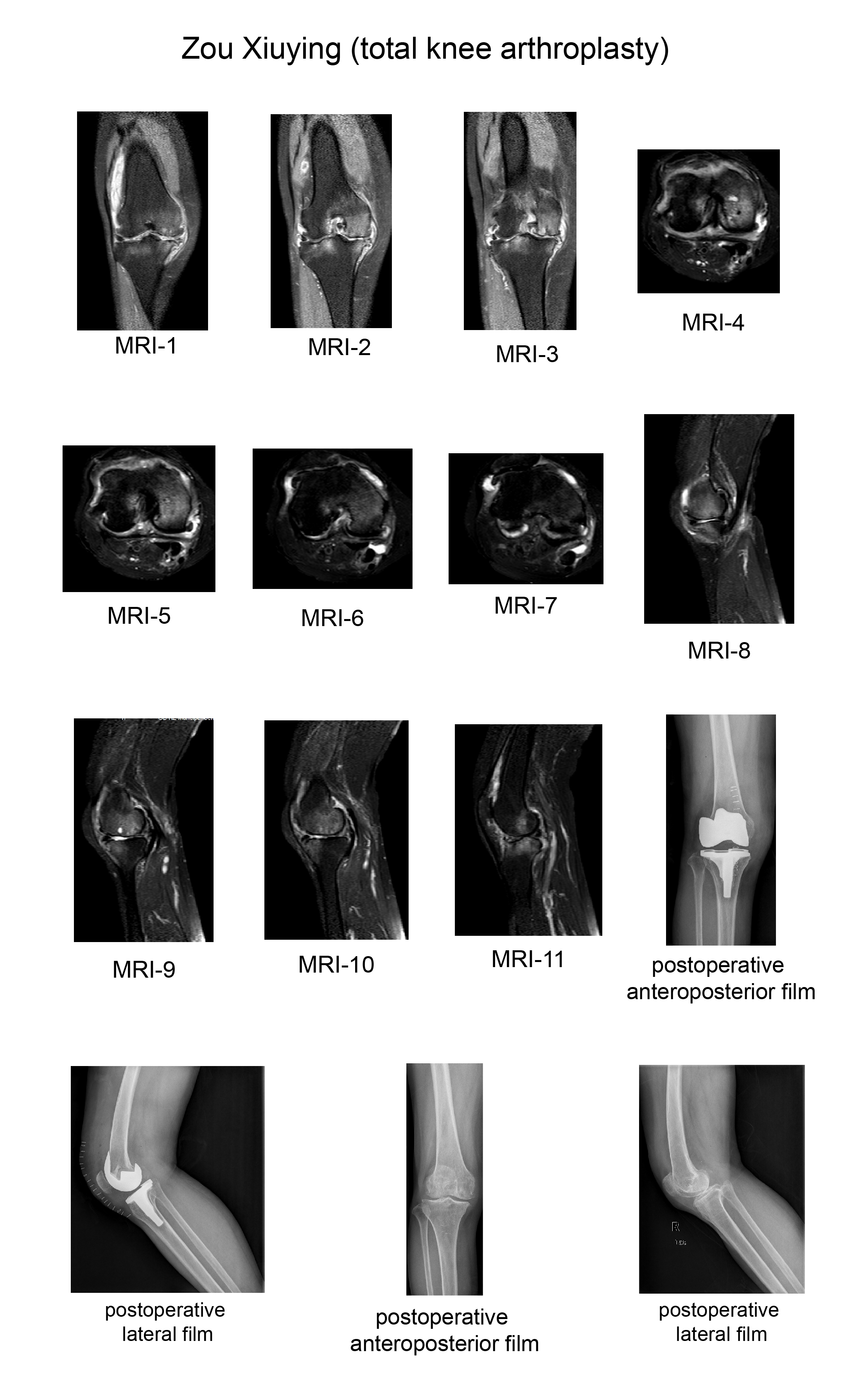

Supplement: Supplementary file 20 — Additional file 20. [file 13018_2020_2070_MOESM20_ESM.jpg]
